# Quantum affective processes for multidimensional decision-making

**DOI:** 10.1038/s41598-022-22855-0

**Published:** 2022-11-28

**Authors:** Johnny K. W. Ho, Johan F. Hoorn

**Affiliations:** 1 School of Design, The Hong Kong Polytechnic University, Hung Hom, Hong Kong; 2 Laboratory for Artificial Intelligence in Design, Hong Kong Science Park, New Territories Hong Kong; 3 Department of Computing, The Hong Kong Polytechnic University, Hung Hom, Hong Kong; 4 Department of Communication Science, VU University Amsterdam, Amsterdam, Netherlands

**Keywords:** Computational science, Human behaviour, Qubits

## Abstract

In modeling the human affective system and applying lessons learned to human–robot interaction, the challenge is to handle ambiguous emotional states of an agency (whether human or artificial), probabilistic decisions, and freedom of choice in affective and behavioral patterns. Moreover, many cognitive processes seem to run in parallel whereas seriality is the standard in conventional computation. Representation of contextual aspects of behavior and processes and of self-directed neuroplasticity are still wanted and so we attempt a quantum-computational construction of robot affect, which theoretically should be able to account for indefinite and ambiguous states as well as parallelism. Our Quantum Coppélia (Q-Coppélia) is a translation into quantum logics of the fuzzy-based Silicon Coppélia system, which simulates the progression of a robot’s attitude towards its user. We show the entire circuitry of the Q-Coppélia framework, aiming at contemporary descriptions of (neuro)psychological processes. Arguably, our work provides a system for simulating and handling affective interactions among various agencies from an understanding of the relations between quantum algorithms and the fundamental nature of psychology.

## Introduction

To design empathic androids that can handle the emotional ambiguity of their users and can simulate mixed feelings themselves, Hoorn et al. developed a fuzzy logic approach to robot affect^1^. Such a robot system should be able to process the affective impulses from its surroundings, in most cases, human affect. To gain a more precise theoretical understanding, we let the robot try to mimic the affective processing system of a human in a fuzzy manner. However, as fuzziness facilitates the simulation of certain affective processes and states, it also has its limitations, which are to be explained later. To overcome the limitations of fuzzy logics and to open up psychological processing to quantum computing, Hoorn et al. developed a quantum understanding of human information processing in terms of mixes of reflective and affective operations^2^, expressed as Bloch vectors^3^, in a sense that information is conceived of as oscillations of electrons that can be superposed, resulting into “mixed states” of reflection and affect, which are described by the probability distributions of the multiple pure states that the oscillations can be in (cf. Raghuvanshi & Perkowski^4^, Yan et al.^5^). The essence of such understanding originates from the basic idea of the quantum that a quantity that is seemingly allowed in one state only (e.g., position, momentum, and energy) may form a single state that may be observed in either capacity with a certain probability.

Why would we want to make the “quantum turn” and not stick to conventional computing when simulating human affect? Since the mid-1990s, a body of literature is building up, proposing that quantum mechanisms are active in human information processing^6,7^. In their review, Schwartz et al. observed that modern physics takes into account psychological decisions in the explanation of causal physical relationships^8^. Reversely, they observed that neuroscientists and psychologists increasingly (should) rely on quantum physics to describe neural processes that are determined by certain structural aspects of the ion channels that are operative in the synapses (the human information “switchboards”). In their seminal work, Hameroff et al. suggested that coherent quantum processes take place in the “microtubules” of brain neurons, controlling the activity of synapses and membranes, underlying occurrences of conscious awareness and decision-making^7^. Schwartz et al. assert that “... contemporary physical theory must in principle be used when analyzing human brain dynamics”^8^.

With its preoccupation with studying phenomena as discrete units, classic science struggles with the contextual aspects of an entity’s behavior and processes (whether in physics or psychology). Psychology may find it difficult to include contextual aspects into its classical probabilistic models, which may be resolved by applying quantum probability theory to handle the dynamics of contextual impact on behaviors^9,10^. In particular, the quantum paradigm facilitates considerations of counterfactual reasoning: “what if the truth should be understood in an alternative way.” A new state of thought, comprising two (or more) perspectives coincides with the quantum realm. Classic probability estimates struggle with probability fallacies such as conjunction and disjunction, as well as irrational behaviors, and order effects^11–19^. By contrast, quantum probability can resolve such paradoxes.

Schwartz et al. criticize contemporary brain science for assuming that measurable physiological data are the final explanation of psychological functions^8^. Apart from the conundrum of “measurement” in quantum physics, these authors point out that contemporary neuropsychology cannot explain what happens during experimentation; how people may “willfully induce brain changes” or employ “self-directed neuroplasticity,” for instance, through training, cognitive re-attribution, or conditioned attentional focus shifts (which may not be intended by the very experiment). Current neuropsychology should incorporate the mathematics of quantum physics to account for human observational bias in the measurement of physical properties of the human brain^8^. The key element added to the dynamics by the quantum formalism is the knowable choices made by human agencies. The classical approach, in contrast, eliminates the causal efficacy of human’s conscious efforts. According to quantum theory, if one attempts to observe a single particle such as an electron traversing a neuron, only one of all the parallel states will be returned (e.g., with electroencephalograms or EEGs). This is well known from Young’s double-slit experiment of electron diffraction. One view on such an observation problem is the property of quantum entanglement. If we take the observer and the observed system as a quantum system, and the observer performs a measurement on the system, the observer presents itself in a particular state by measuring itself (in the basis of the observer), and then a measurement is performed on the observed system (in its basis) given the state of the observer. In other words, the observer and the observed system are entangled, in a sense that the state of the observed system depends on and is relative to the state of the observer.

If we should believe the neurologists and psychologists, then the firing frequencies of electrons carry information around the human brain, over a trajectory, say the ion shafts in the nervous system, which allow neurons to generate action potentials. Such processes are susceptible to quantum dynamics too, including the superposition of an electron’s wave function over different locations in the brain – perhaps even other body parts, or perhaps other people? If so, will superposed electrons explain the dynamics of human information processing, in particular of affect and reflection operating on the same piece of information in parallel?

We will attempt the modeling of the ambiguity or “polyvalence” of human emotions, such that a robot could simulate them in a human-like fashion. Raghuvanshi et al. introduced the “quantum sphere of emotions” in the Bloch sphere derived from Plutchik’s wheel^4^. The active–passive dimension of human behavior and the positive–negative emotions form the *xy*-plane at a value of *z*, denoting emotional intensity. In Raghuvanshi’s view, different emotions correspond to different regions on the Bloch-sphere surface as indicated by the phase of the quantum state, which allows for an ensemble of emotions rather than just one. Yan et al. represented human emotions using the Bloch sphere derived from the pleasure–arousal (PA) plane in which emotions are associated with different regions^5^, similar to the two-dimensional circle representation of activation (arousal) and valence^20–22^. With the *z*-axis representing emotion intensity, a point on the Bloch sphere defined by a qubit representing the length of the Bloch vector depicts the emotional ambiguity. Represented like this, the emotion “surprise” may have different levels of liveliness and may have pleasurable as well as unpleasable aspects concurrently. Yan et al. propose a number of variants. Their earlier work proposed that the PA plane is the *xy*-plane enclosed by the equator^5^. Later, discretization of the PA plane using a qubyte was proposed^23^. In recent work, the angular position is modeled as the polar angle of the qubit state^24^, and the information of emotion intensity is included comprising of $2^n$ levels, using *n* qubits incorporated with the Plutchik wheel^25^. Lately, a 3-qubit representation was proposed by Yan et al. based on the pleasure–arousal–dominance (PAD) model, each denoting the pleasure, arousal, and dominance dimension^26^. Apart from emotion representation, these authors also proposed the correspondence between human behavior and quantum modeling^4^, algorithms for quantum emotions in a time domain^5,23,25,26^, and in a multi-robot domain^24^. Despite the various representations and paradigms of quantum emotions, an underlying contextualized process of affect for those quantum emotions is still missing.

Earlier, we worked on the affective system coined Silicon Coppélia^1^. Silicon Coppélia is equipped with fuzzy logic with a complete and elaborated implementation of interpreting observed features, comparing those with goals, and evaluating the decision to an action based on the use intentions and level of engagement^1^. Emotion-regulation strategies are present inside the Silicon Coppélia system to resemble cognitive control over sometimes too intense affective responses. In Silicon Coppélia, affective and cognitive processes balance each other, for instance, when showing empathy (taking a cognitive perspective and adding positive affect). The concurrent affective and reflective processes try to mimic the neurological dynamics inside the human brain. On a neurological level, information is forwarded by firing frequencies of electrons. The thalamus works as a semi-transparent mirror: Information runs directly to the amygdala; psychologists would term this “affective processing”; and concurrently that same information splits off to the neocortex and only then enters the amygdala, which psychologists would regard as “reflective processing”^27–29^. It follows that the information entering the amygdala is present in more than one state all at the same time. Information would directly enter the amygdala to detect friend or foe and the detour through the neocortex would add to the feeling of being involved with the agency or feeling friendship for it. In sum, the Silicon Coppélia system possesses both psychological and neurological compatibility for quantum processes, which is promising for transitioning it to a system for quantum-information processing and computing, Quantum Coppélia for short. Previously, we cited the quantum-consciousness literature^7^ because on the affective front, there are few papers that delve into affective processes^4,5,24,25^. Looking into quantum-consciousness seems the next best thing, particularly to find a biological substrate for assumed quantum phenomena in the brain^7^. By that, however, we do not intend to equate psychological affect with “emotions” or “feelings,” nor do we mean that affect is necessarily conscious: Some affective processes that produce emotions happen on reflex (i.e., fear); they are evolutionary hard-coded and may happen unconsciously^30^.

Quantum Coppélia (Q-Coppélia) attempts to provide a conceptual framework of an affective system for quantum computation, which is sufficiently specific for processing real observations, based on the Silicon Coppélia model^1^. In Section “Theory” of this article, selected quantum properties are highlighted as the foundation of Q-Coppélia, followed by discussions of certain building blocks of the quantum circuits. Then, with a brief review of the Silicon Coppélia system in Section S1, the construction of Q-Coppélia is provided in Section “The Quantum Coppélia model”, component by component, each corresponding to the original Silicon Coppélia system. In Section “Discussion”, we compare Q-Coppélia with Silicon Coppélia in various aspects: Q-Coppélia can differentiate concurrently occurring affective states and the subsequent decision to execute a certain behavior. That decision usually is observed as the result of one type of affect alone; although such a decision does not necessarily reflect all the affective states that people actually have in mind. The quantum nature of Q-Coppélia allows for probabilistic decisions that are not present in Silicon Coppélia, representing the indeterminism of human behavior. It also naturally embeds the capability of parallel processing, in line with parallel distributed neural processes. Q-Coppélia generalizes further the representation of ambiguity and provides a conceptually distinct understanding of a scale of affect as states of linear independence. Finally, a comparison of Boolean convention in Silicon Coppélia and Q-Coppélia is noted, revealing a possible arbitrary choice of affective and behavioral patterns. In demonstrating the quantum transition of a classical affective processing system, our work reveals a contextual understanding of the relations between the quantum algorithms and the fundamental nature of psychology, facilitating further explorations of contextual quantum-affective systems.

## Theory

**Figure 1:**
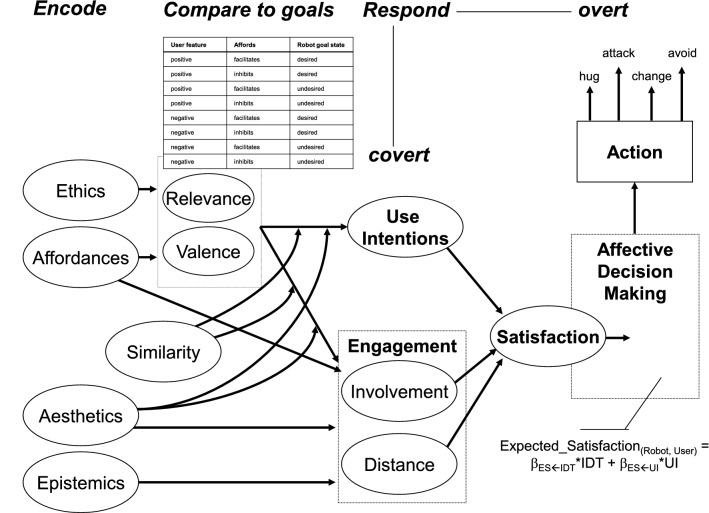
Dependencies in Silicon Coppélia^32^. Curved arrows indicate interaction effects. *IDT* involvement–distance trade-off, *UI* use intentions.

In earlier work on affective processing, Hoorn et al. discerned a feature-encoding phase, a comparison phase, and a response phase, using fuzzy logic to represent a robot’s response (Fig. 1)^1^. In Fig. 1, Silicon Coppélia encodes the observed features of the target agency, and appraises them in various domains (e.g., *Ethics*) for comparison with the goals and concerns of the robot, determining *Relevance* and *Valence*. The response takes into account intentions to work with the target agency and the level of engagement with the target (i.e., *Involvement* and *Distance*). Silicon Coppélia offers a full mathematical account, implemented in Ptolomy^31^. In the remainder of this paper, a brief review of the model is given in Section S1, together with an attempt to better systematize the use of mathematical language for deployment in the quantum-computing algorithms, presented thereafter.

### Circuit components for quantum logic

#### Dimension scales of state variables and initialization

**Figure 2:**
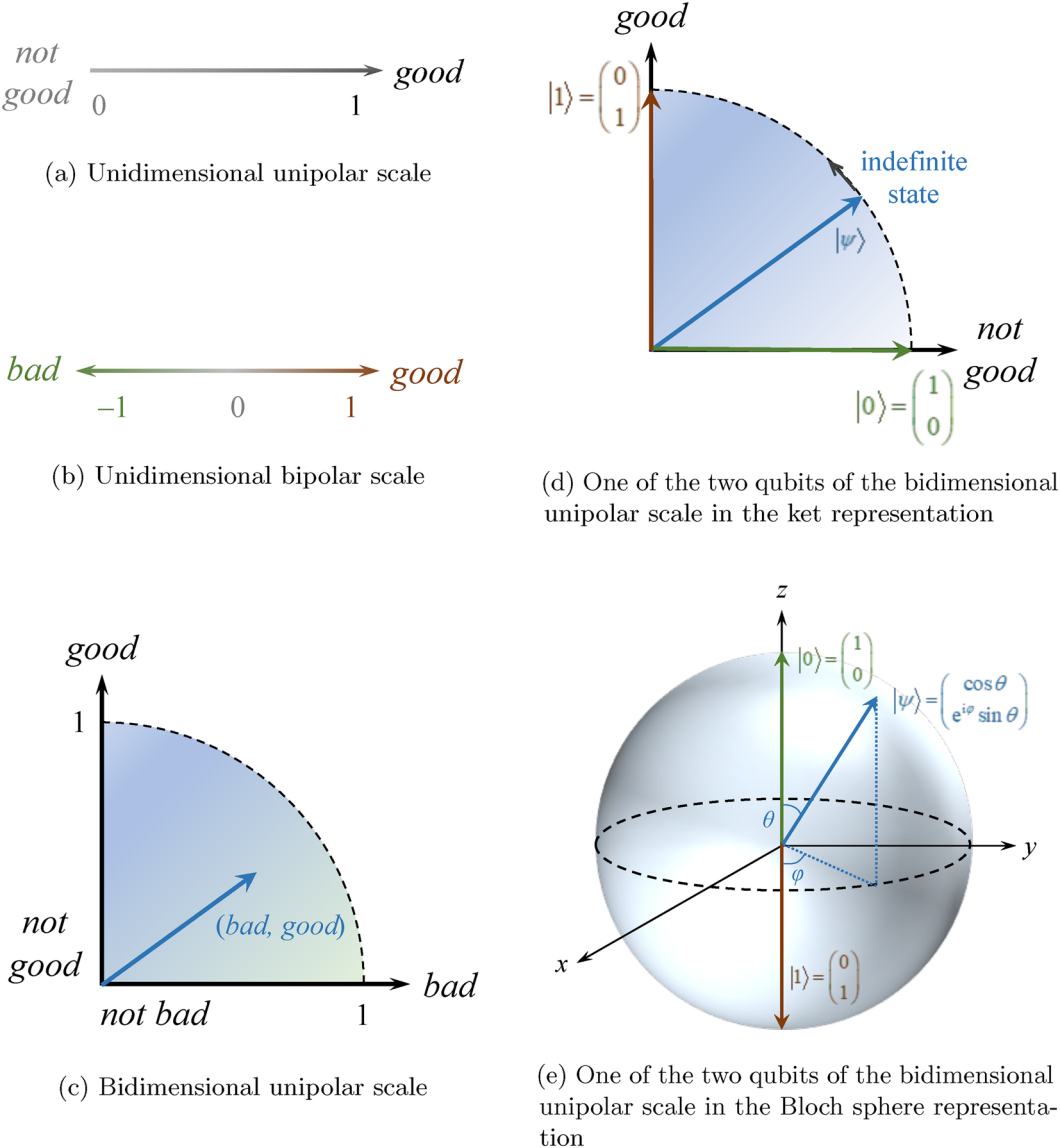
Representation of different scales. The two appraisal labels of Ethics, *good* and *bad*, are used as an example. Unidimensional unipolar scales can be found in the weight parameters. Vectors not reaching the surface of the Bloch sphere indicate mixed states.

The dimensions and variables in the Silicon Coppélia system may come as unidimensional bipolar scales and bidimensional unipolar scales. A unipolar scale (e.g., unidimensional, Fig. 2a) defines an attribute of the extent of a quality, such as how good, bad, or happy. A unidimensional bipolar scale (Fig. 2b) consists of a single psychometric scale of which the ends are usually interpreted as near-opposite quantities, such as happy and sad, good and evil, etc. It is usually quantified with a range of positive and negative numbers, e.g., $[-1,1]$. A bidimensional unipolar scale (Fig. 2c), on the other hand, consists of two scales that are usually interpreted as a complement of each other. The ends of the two scales indicate “with it” and “without it.” It is usually quantified with a set of ordered pair with non-negative numbers, e.g., $\left\{ \left( x,y\right) \vert \ x,y \in [0,1]\right\}$. Complement scales are linearly independent, i.e., one scale cannot be expressed equivalently as the other by multiplying it by a constant. In practice, these scales are often treated as orthogonal, between which there is no contextual intersection. Therefore, bidimensional unipolar scales are a generalization of unidimensional bipolar scales in the sense that bidimensional unipolar scales treat the quantities as separate dimensions and allow the coexistence of the complement pair. The unidimensional bipolar scale attributes either good or bad, beautiful or ugly, to a feature, whereas the bidimensional unipolar scale allows it to contain both simultaneously (something good in the bad).

In Q-Coppélia, a psychometric scale is represented by a quantum state denoted by a ket ($|{\cdots } \rangle$), as shown in Fig. 2d. The scale is a two-state system, consisting of a basis of two vectors. A choice of a two-state system could mimic human behavior that results from the decision for action, representing one of the multiple emotions concurrent in the mind^4^, i.e., overt behavior shows a level of “certainty” about one’s vague or ambiguous feelings that covertly are actually present. In choosing $|{0} \rangle$ and $|{1} \rangle$ as the computational basis, these kets form a complete set of orthogonal basis in the Hilbert space, i.e.,  any single ket can be represented by some linear combination of the basis vectors. When $|{0} \rangle$ and $|{1} \rangle$ form a scale, $|{1} \rangle$ is defined as “full of something” (full scale) and $|{0} \rangle$ as “absence of something.” If one interprets physically that different scales are governed by different parts of the brain, $|{1} \rangle$ indicates the presence of an action potential and that a certain part of the brain is activated (being certain of the appraisal once measured so). A unidimensional scale is modeled as one qubit and a bidimensional unipolar scale is modeled as two qubits, each of them denoting one scale of the complement pair, e.g., *good* and *bad*. Whereas a scale is normally represented by a range of scalar, here it is represented by a linear combination of the basis vectors and visualized in a Bloch sphere (Fig. 2e). Therefore, different kets are (mathematically) regarded as distinct states of no alikeness (since ${\langle }{0}|{1}{\rangle } = {\langle }{1}|{0}{\rangle } = 0$, states not linearly dependent cannot be compared as “more” or “less”). The advantage of having a complete set of states using an orthonormal basis is that it allows the unique description of all states of interest. Likewise, parallel possibilities of $|{0} \rangle$ and $|{1} \rangle$ do not interfere with each other. The change in the scale is denoted as a transformation of the ket from one to another, instead of the classical way of algebraic addition or subtraction. In other words, the scales do not possess properties of reinforcement and cancellation (e.g., “very happy” and “no feeling of happiness” are two distinct states; they cannot be regarded as “fairly happy on average”). Because every state is represented by a ket, indefinite states (neither $|{0} \rangle$ nor $|{1} \rangle$ but a superposition of them) and statistical mixtures of states could be denoted by a qubit, and all states may be processed in one computation, known as quantum parallelism. This approach would simulate simultaneous processing of human affect.

In general, a pure state seen as an indefinite state formed by the superposition of the basis vectors is interpreted as an intermediate scale. For example, a quantum state $\frac{|{0} \rangle +|{1} \rangle }{\sqrt{2}}$ of *good* as part of the complementary *good*/*bad* pair on a bidimensional unipolar scale is a vague state both “with *good*” and “without *good*” with equal probabilities. With a similar construction for *bad*, a bidimensional unipolar scale is formed. For both scales, a measurement of the qubit returns either $|{0} \rangle$ or $|{1} \rangle$.

A quantum state contains both information of probability amplitude and phase. A pure state may be written as

1 $$\left| \psi \right\rangle = \cos\frac{\theta}{2} \left| 0 \right\rangle + \mathrm{e}^{\mathrm{i}\varphi} \sin\frac{\theta}{2} \left| 1 \right\rangle$$

where $\theta$ and $\varphi$ represent the polar angle and the azimuthal angle of the Bloch sphere, respectively. The pure state $|{\psi } \rangle$ is a point on the Bloch sphere surface. Such a general state may be initialized from $|{0} \rangle$ by

2 $$\begin{aligned} |{\psi } \rangle&= \begin{bmatrix} \cos \frac{\theta }{2} \\ \mathrm {e}^{\mathrm {i}\varphi } \sin \frac{\theta }{2} \end{bmatrix} \nonumber \\&= \begin{bmatrix} \cos \frac{\theta }{2} &{} \sin \frac{\theta }{2} \\ \mathrm {e}^{\mathrm {i}\varphi } \sin \frac{\theta }{2} &{} \mathrm {e}^{\mathrm {i}\varphi } \cos \frac{\theta }{2} \end{bmatrix} \begin{bmatrix} 1 \\ 0 \end{bmatrix} \nonumber \\&= \begin{bmatrix} 1 &{} 0 \\ 0 &{} \mathrm {e}^{\mathrm {i}\varphi } \end{bmatrix} \begin{bmatrix} \cos \frac{\theta }{2} &{} -\sin \frac{\theta }{2} \\ \sin \frac{\theta }{2} &{} \cos \frac{\theta }{2} \end{bmatrix} \begin{bmatrix} 1 \\ 0 \end{bmatrix} \nonumber \\&= \mathrm {P}\left( \varphi \right) \mathrm {R}_y \left( \theta \right) |{0} \rangle \end{aligned}$$

where P and $\hbox {R}_y$ are the phase gate and $\hbox {R}_y$ gate, respectively. In other words, an arbitrary pure state may be initialized by applying $\mathrm {R}_y \left( \theta \right)$ and $\mathrm {P} \left( \varphi \right)$ sequentially to $|{0} \rangle$. Initialization is performed when specifying parameters and any predefined states.

#### Logical and register operations and register-triggered logics

**Figure 3:**
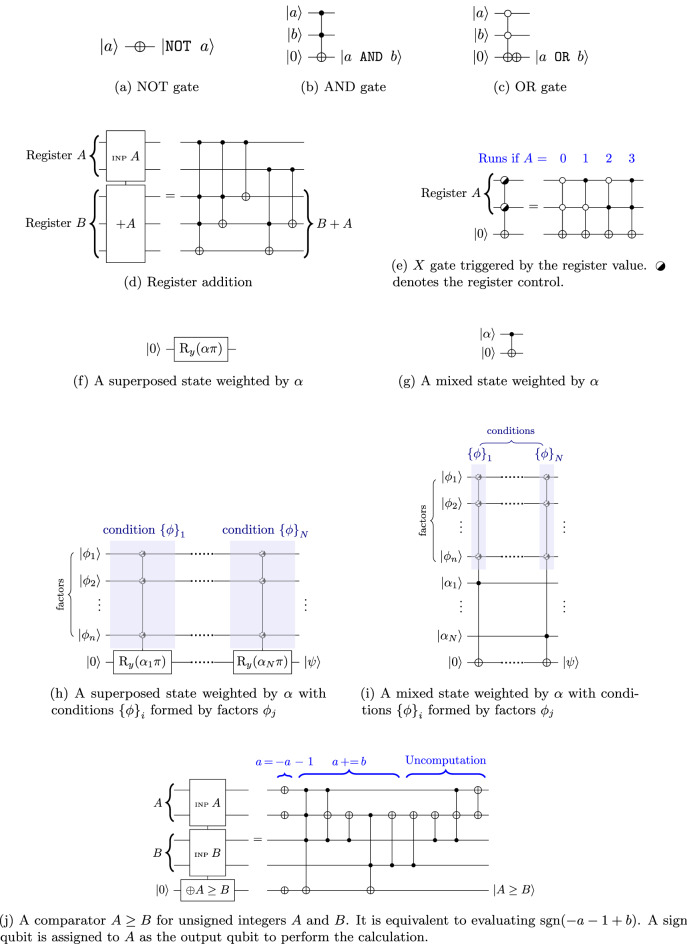
Some building blocks of quantum gates in the “little endian representation”. The symbol  may represent a $|{0} \rangle$-triggered control ($\bullet$), a $|{1} \rangle$-triggered control ($\circ$) or nothing, depending on the details of the condition.

The affective process described by Silicon Coppélia involves numerous fuzzy not, and and or operations, which correspond to the X gate, generalized Toffoli AND gate, and the generalized Toffoli OR gate, respectively (Fig. 3a–c). When composite logic is involved, ancilla qubits are used to store the intermediate results. For processes involving generalized controlled-NOT operations only, uncomputation is performed after the logic evaluation so that the ancilla qubits can be recycled. Uncomputation repeats the operations that entangled the ancilla qubits but in a reverse sequence except for the operations that produce the final output.

Quantum registers (qubytes) may also be involved in the operations of quantum computing. One commonly used qubyte operation is addition (Fig. 3d). Quantum algorithms may sometimes be conditional to the value of the qubytes. The conditional expressions are accomplished by setting the qubyte as the control register, where the value for triggering the operation is labeled with the $|{1} \rangle$–conditioned control ($\bullet$), and $|{0} \rangle$–conditioned control ($\circ$) for the others (Fig. 3e).

#### Superposition and mixed states manipulations

Whereas both superposition and mixed states denote some states not exactly $|{0} \rangle$ or $|{1} \rangle$, they are different in nature. A pure state, being a representation of vagueness, denotes one’s single indefinite affective state. A pure affective state contains no uncertainty for the affect of the robot (but only the decision upon measurement depending on the basis of the measurement). On the other hand, a mixed state reveals that one’s affective state is ambiguous, i.e., one may be in a pure state of (certain) $|{\psi _1} \rangle$ or $|{\psi _2} \rangle$, but that cannot be known until an observation is made or a measurement is done. Suppose that one’s evaluated positive valence $\bigl|{V^{(k)}} \bigr\rangle$ towards a certain feature is not extremely strong. In modeling positive valence as a two-state system where $\bigl|{V^{(k)}} \bigr\rangle = |{0} \rangle$ and $|{1} \rangle$, which represent absolutely no and absolutely strong positive valence, respectively, the state of “I feel some positive valence” would be expressed in a quantum superposition of $|{0} \rangle$ and $|{1} \rangle$, i.e., as vagueness. Upon decision-making (measurement), the state is forced to collapse to either strong or no positive valence. On the other hand, the agency may perceive “mixed feelings” of, for example, extremely strong positive valence as well as “nothing” due to, for instance, concurrent distinct perspectives. This is ambiguity. Then the positive valence evaluation produces a mixed state. Unlike superposition where the state of strong or no positive valence collapses from the indefinite state upon measurement, the two extremes in the mixed state exist *before* the decision-making process. The decision-making process only picks one of the two extremes as a probabilistic selection of perspectives followed by measurement of the chosen perspective, if applicable, instead of merely collapsing a vague state of superposition. As a remark of generalization, a mixed state may be composed of different pure states of superposition.

In terms of quantum computation, superposition is established by rotation of the state about the *x*- or *y*-axis in the Bloch sphere, or by the Hadamard gate. A mixed state is made by the entanglement of states typically using controlled operations (e.g., CNOT). Therefore, if the algorithm aims at producing a single state as a whole given certain conditions, a rotation gate (superposition) should be used. On the other hand, if the algorithm attempts to produce ambiguity, saying that multiple possibilities of pure states are possible, a controlled operation (mixed state) should be used.

A state that is partially affected by a factor may be characterized by a weight factor. For an endomorphic resultant state possessing the same purity as the original state, the operation can be accomplished with a rotation of an angle of $\alpha \mathrm {\pi }$ (Fig. 3f), called the *rotation formalism* here. The resultant state would be one of certain superposition. The angle of rotation equals $\mathrm {\pi }$ if the factor has absolute dominance, i.e., $\alpha = 1$. In other words, $\alpha$ describes the degree of superposition or the degree of vagueness. On the other hand, a mixed state can be generated from a weight factor by connecting the target qubit to a control qubit $|{\alpha } \rangle$ which represents the weight using the CNOT gate (Fig. 3g), termed the *entanglement formalism*. Zero weight is given by a control qubit that equals $|{0} \rangle$, and equals $|{1} \rangle$ for full dominance. The mixed or superposed control qubit would retain the target state with its $|{0} \rangle$ component and toggles the target qubit state with its $|{1} \rangle$ component, according to the probability amplitude of the two components. This results in a mixed state of the target qubit. Effectively, under a controlled operation, the target qubit breaks down into its constitute bases of the gate and undergoes gate operations depending on the state components. Therefore, mixed states do not have a well-defined basis and are subject to the basis of the measurement, which stands in contrast to a pure state, which can be described as “it is a definitive state in a particular basis”.

In both approaches, additional conditions may also be included in the algorithm as control qubits. In general, a resultant state may be generated conditionally from multiple factors, each contributing with a particular weight. Suppose that a state $|{\psi } \rangle$ initially set as $|{0} \rangle$ is generated by *N* conditions from *n* factors $|{\phi _i} \rangle$ ($i = 1,2,\cdots ,n$), where each condition carries a weight $\alpha _j$ ($j=1,2,\cdots ,N$) such that $\sum _j^N \alpha _j = 1$. Additionally, suppose that the state is $|{1} \rangle$ if all conditions are met. Then each condition may be represented by a set of control qubits at the relevant wires of factor $|{\phi _i} \rangle$. For the entanglement formalism, the implementation is denoted as a series of the generalized Toffoli gates, each involving a set of qubits of the relevant factors $|{\psi _i} \rangle$ (Fig. 3i). For the rotation formalism, the operation is modeled as successive conditional rotations of the state (Fig. 3h). The controlled rotation is analogous to the rule strength in fuzzy logic in the sense that the state of the control qubit determines the expectation of the measurement outcome, corresponding to the output value of fuzzy logic. The resultant state may be written as:

3 $$\begin{aligned} |{\psi } \rangle = \text {C-R}_y \left( \alpha _N \mathrm {\pi },\{\phi \}_{\!N}\right) \;\cdots \; \text {C-R}_y \left( \alpha _2 \mathrm {\pi },\{\phi \}_{\!2}\right) \ \text {C-R}_y \left( \alpha _1 \mathrm {\pi },\{\phi \}_{\!1}\right) \; |{0} \rangle \end{aligned}$$

where C-$\hbox {R}_y\left( \theta _j,\{\phi \}_{\!j}\right)$ is the controlled $\hbox {R}_y$ rotation for the $j{\text {th}}$ condition subject to a set of factors $\{\phi \}_j$ containing respective $\phi _i$ (one condition can be made up of multiple factors). Due to the use of the control qubits, the purity of the state would be changed, leading generally to a mixed state. Nonetheless, as discussed in Section “Vagueness and ambiguity”, the purity of such a state is generally higher than the mixed state counterpart.

#### Decision-making process from individual qubit (affective) states

According to Section S1.3.3, the decision-making process involves determination of the index that gives the greatest value (e.g., feature *k* for the expected satisfaction $S^{(k)}$ [Eq. (S19)], and choice of action $i^{(k_{\max })}$ for the expected satisfaction for the action [Eq. (S22)]. Given that every feature has its own qubit of expected satisfaction, a comparison has to be done to find the qubit that gives the greatest value. The output is a qubyte that stores the value of feature *k* possessing the greatest expected satisfaction. However, there are two complications. For one, the input is *separate* (uncorrelated), *unordered* qubits (without grouping them into a register) of satisfaction values that do not indicate (no information of) the value of *k*. Therefore, the system cannot match the satisfaction values to the feature, which is the output of the comparison. For the other, when quantum logic is employed, superposed or mixed states introduce various possibilities of which the expected satisfaction is the greatest, each associated with a probability. Similar situations happen for the determination of the resulting action tendencies. Therefore, a representation of quantum integers is needed, i.e., qubytes that can store integers in a probabilistic manner, denoting the feature *k* of the greatest satisfaction value.

**Figure 4:**
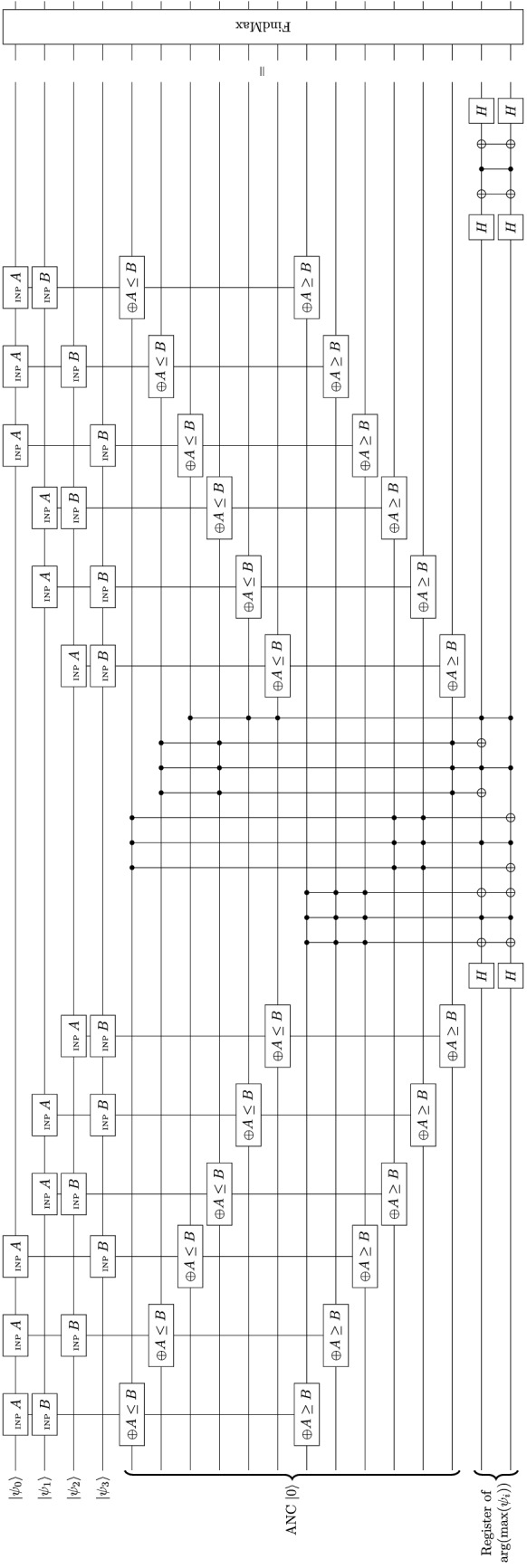
Example algorithm for generating a qubyte that indexes the maximum of 4 qubits $|{\psi _i} \rangle$ ($i=0,1,2,3$).

The maximum search algorithm consists of (1) comparing the values (finding the maximum) and storing the results in ancilla qubits and (2) converting the comparison results to a qubyte (finding the argument). Both could be substituted by more advanced constructions. Here, Fig. 4 shows an example circuit of finding which out of the four qubits gives the maximum value. In a two-state system, defining $|{1} \rangle$ is greater than $|{0} \rangle$, $P^4_2 = 12$ comparisons of $A \ge B$ are made, and every comparison result is stored in an ancilla qubit (so 12 ancilla qubits are needed). During coding, $C^4_2 = 6$ pairs of qubits are chosen. For every pair, the two qubits are assigned to be *A* and *B*, which are then fed into the at-most gate ($\oplus A \le B$) and the at-least gate ($\oplus A \ge B$) targeted to the ancilla qubits. A typical quantum algorithm for the comparator is shown in Fig. 3j. The ancilla qubit is toggled if the boolean returns True. Therefore, the ancilla qubits are in general mixed states and store whether one qubit is greater than the other. Mixed states arise because of the vagueness or ambiguity of the states. For example, for two states $|{\psi _1} \rangle = \alpha _0 |{0} \rangle _1 + \alpha _1 |{1} \rangle _1$ and $|{\psi _2} \rangle = \beta _0 |{0} \rangle _2 + \beta _1 |{1} \rangle _2$, the comparison involves all combinations of $|{n} \rangle _1$ and $|{n'} \rangle _2$ ($n,n'=0,1$) associated with a probability of $|{\alpha _n^*\beta _{n'}} |^2$. The comparison results are converted to a qubyte that allows entanglement. Every qubit of the expected satisfaction corresponds to a qubyte value. The value of the qubyte is the value of the feature of the greatest satisfaction. The qubyte is first set into superposition. Then for each qubyte value, a phase flip is carried out if the corresponding qubit of satisfaction is the maximum, i.e., the conjunction of 3 Boolean expressions that the one of interest is greater than all the other three. Then the phase factor represents whether the feature gives the greatest satisfaction. After that, a mirror operation is performed to turn the phase information into amplitude information. The amplitude represents the relative phase contrast between the qubyte values as if normalizing the probabilities of occurrence. Meanwhile, the ancilla qubits are uncomputed for later use. Finally, the entire qubyte is measured to obtain a classical byte whose value depicts the feature of the greatest expected satisfaction.

It should be noted that the above process is carried out in a parallel and probabilistic manner. In general, a statistical mixture of states (mixed states of satisfaction) is processed. For each pure state involved, the qubit that gives the greatest satisfaction is different, carrying a certain probability. Therefore, during the comparison, there are phase flips for various qubits with the associated probabilities. The operation works naturally in parallel. In other words, various features, instead of only one conditionally, would possess the maximum satisfaction with their respective probabilities. The information stored in the phase factor represents whether the feature bears the maximum satisfaction (flipped if it does, unflipped if not). Under the measurement, the system “chooses” the feature of the maximum satisfaction according to the probability distribution.

## The Quantum Coppélia model

In this section, the construction of Q-Coppélia is revealed component by component, each corresponding to a particular segment of the Silicon Coppélia system (see Section S1). In each section, the corresponding portion of the Silicon Coppélia system is indicated for tracking. Tables S1–S5 list the quantum transition of the variables. Variables of unidimensional bipolar and bidimensional unipolar scales are represented by a pair of qubits as two-state systems of complementary properties. The construction of Q-Coppélia uses the building blocks mentioned in Section “Theory”. All ancilla qubits, unless specified, are initially $|{0} \rangle$. Similar to Silicon Coppélia, the framework of Q-Coppélia follows modular programming, and thus each part could be replaced by a more sophisticated algorithm without disturbing the overall framework and flow of information.

### Encoding features

The algorithms discussed in this section refer to the processes in Section S1.1. The qubits involved in this section are listed in Table S1. Every feature is interpreted with some weight indicating its dominance in the agency’s interpretation. The weight for each feature is assigned to a qubit $\bigl|{w^{(k)}} \bigr\rangle = w^{(k)}_0 |{0} \rangle + w^{(k)}_1 |{1} \rangle$. In addition, since parallel perception of both sides of an appraisal variable is possible (e.g., someone’s eyes may be perceived concurrently as beautiful and somewhat unattractive), the appraisal variables are treated as bidimensional unipolar scales, having their own qubits of pre-defined appraisal weights $\bigl|{d^{(k)}_{l}} \bigr\rangle = d^{(k)}_{l\!,0} |{0} \rangle + d^{(k)}_{l\!,1} |{1} \rangle$ ($l \in {\mathscr {D}}$), and perceived weight $\bigl|{p^{(k)}_{l}} \bigr\rangle$, which is initialized to be $|{0} \rangle$. Then, with $\bigl|{w^{(k)}} \bigr\rangle$ and $\bigl|{d^{(k)}_{l}} \bigr\rangle$ initialized as the input parameters using Eq. (2) or fed from other outputs, a quantum formulation for Eq. (S1) is modeled using a Toffoli gate such that

4 $$\begin{aligned} \Bigl|{p^{(k)}_{l}} \Bigr\rangle \Bigl|{d^{(k)}_{l}} \Bigr\rangle \Bigl|{w^{(k)}} \Bigr\rangle &= \Bigl|{0 {\texttt{\ XOR\ }} \bigl( d^{(k)}_{l} {\texttt {\ AND\ }} w^{(k)} \bigr) } \Bigr\rangle \Bigl|{d^{(k)}_{l}} \Bigr\rangle \Bigl|{w^{(k)}} \Bigr\rangle \nonumber \\&= \begin{bmatrix} 1\vphantom{d^{(k)}_{l,\!0}} &{} 0 &{} 0 &{} 0 &{} 0 &{} 0 &{} 0 &{} 0 \\ 0\vphantom{d^{(k)}_{l,\!0}} &{} 1 &{} 0 &{} 0 &{} 0 &{} 0 &{} 0 &{} 0 \\ 0\vphantom{d^{(k)}_{l,\!0}} &{} 0 &{} 1 &{} 0 &{} 0 &{} 0 &{} 0 &{} 0 \\ 0\vphantom{d^{(k)}_{l,\!0}} &{} 0 &{} 0 &{} 0 &{} 0 &{} 0 &{} 0 &{} 1 \\ 0\vphantom{d^{(k)}_{l,\!0}} &{} 0 &{} 0 &{} 0 &{} 1 &{} 0 &{} 0 &{} 0 \\ 0\vphantom{d^{(k)}_{l,\!0}} &{} 0 &{} 0 &{} 0 &{} 0 &{} 1 &{} 0 &{} 0 \\ 0\vphantom{d^{(k)}_{l,\!0}} &{} 0 &{} 0 &{} 0 &{} 0 &{} 0 &{} 1 &{} 0 \\ 0\vphantom{d^{(k)}_{l,\!0}} &{} 0 &{} 0 &{} 1 &{} 0 &{} 0 &{} 0 &{} 0 \end{bmatrix} \begin{bmatrix} d^{(k)}_{l\!,0} w^{(k)}_0 \\ d^{(k)}_{l\!,0} w^{(k)}_1 \\ d^{(k)}_{l\!,1} w^{(k)}_0 \\ d^{(k)}_{l\!,1} w^{(k)}_1 \\ 0\vphantom{d^{(k)}_{l\!,1}} \\ 0\vphantom{d^{(k)}_{l\!,1}} \\ 0\vphantom{d^{(k)}_{l\!,1}} \\ 0\vphantom{d^{(k)}_{l\!,1}} \end{bmatrix} \nonumber = \begin{bmatrix} d^{(k)}_{l\!,0} w^{(k)}_0 \\ d^{(k)}_{l\!,0} w^{(k)}_1 \\ d^{(k)}_{l\!,1} w^{(k)}_0 \\ 0\vphantom{d^{(k)}_{l\!,1}} \\ 0\vphantom{d^{(k)}_{l\!,1}} \\ 0\vphantom{d^{(k)}_{l\!,1}} \\ 0\vphantom{d^{(k)}_{l\!,1}} \\ d^{(k)}_{l\!,1} w^{(k)}_1 \end{bmatrix}. \end{aligned}$$

**Figure 5:**
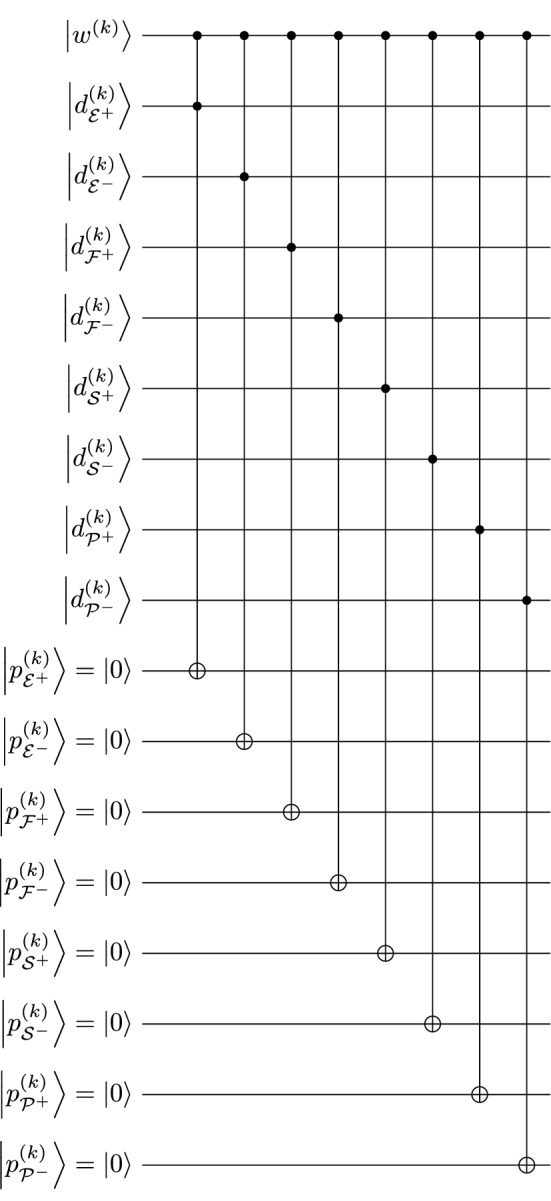
Quantum algorithm for the feature encoding process, corresponding to Eq. (S1).

The density matrix $\bigl|{p^{(k)}_{l}} \bigr\rangle \bigl\langle {p^{(k)}_{l}} \bigr|$ as the partial trace of the density matrix corresponding to the state in Eq. (4), together with the normalization condition ${{\,\mathrm{Tr}\,}}\!\bigl (|{\psi } \rangle \langle {\psi } |\bigr ) = 1$, is

5 $$\begin{aligned} \Bigl|{p^{(k)}_{l}} \Bigr\rangle \Bigl\langle {p^{(k)}_{l}} \Bigr| =&{{\,\mathrm{Tr}\,}}_{0,1} \left( \Bigl|{p^{(k)}_{l}} \Bigr\rangle \Bigl|{d^{(k)}_{l}} \Bigr\rangle \bigl|{w^{(k)}} \bigr\rangle \bigl\langle {w^{(k)}} \bigr| \Bigl\langle {d^{(k)}_{l}} \Bigr| \Bigl\langle {p^{(k)}_{l}} \Bigr| \right) \nonumber \\ =&\begin{bmatrix} \left|{d^{(k)}_{l\!,0}} \right|^2 + \left|{d^{(k)}_{l\!,1} w^{(k)}_0} \right|^2 &{} 0 \\ 0 &{} \left|{d^{(k)}_{l\!,1} w^{(k)}_1} \right|^2 \end{bmatrix} \nonumber \\ =&\left( 1-\left|{d^{(k)}_{l\!,1} w^{(k)}_1} \right|^2\right) |{0} \rangle \langle {0} | + \left|{d^{(k)}_{l\!,1} w^{(k)}_1} \right|^2 |{1} \rangle \langle {1} | \end{aligned}$$

with a purity $1-\bigl|{d^{(k)}_{l\!,1} w^{(k)}_1} \bigr|^2 + \bigl|{\bigl( d^{(k)}_{l\!,1}\bigr) ^2\bigl( w^{(k)}_1\bigr) ^2} \bigr|^2 \in \left[ \frac{1}{2},1\right]$, characterizing ambiguity. In the Bloch sphere representation [Eq. (1)], the maximum ambiguity occurs with a purity of 3/4 when $\sin \bigl( \frac{1}{2}\theta ^{(k)}_{l\!,\mathrm {d}}\bigr) \cos \bigl( \frac{1}{2}\theta ^{(k)}_{l\!,\mathrm {w}}\bigr) = \frac{1}{2}$, where $\theta ^{(k)}_{l\!,\mathrm {d}}$ and $\theta ^{(k)}_{l\!,\mathrm {w}}$ are the polar angles for $\bigl|{d^{(k)}_{l}} \bigr\rangle$ and $\bigl|{w^{(k)}} \bigr\rangle$, respectively. For an observable $${\hat{A}} = \begin{bmatrix} 0 &{} 0 \\ 0 &{} 1 \end{bmatrix}$$, which gives ${\hat{A}} |{n} \rangle = n|{n} \rangle$, the corresponding value of $p^{(k)}_{l}$ can be estimated by $\bigl\langle {\hat{A}} \bigr\rangle = \bigl|{d^{(k)}_{l\!,1} w^{(k)}_1} \bigr|^2$, consistent with Eq. (S1). Figure 5 shows the entire feature-encoding process. Table S1 summarizes the qubits involved in the calculation.

### Relevance

**Figure 6:**
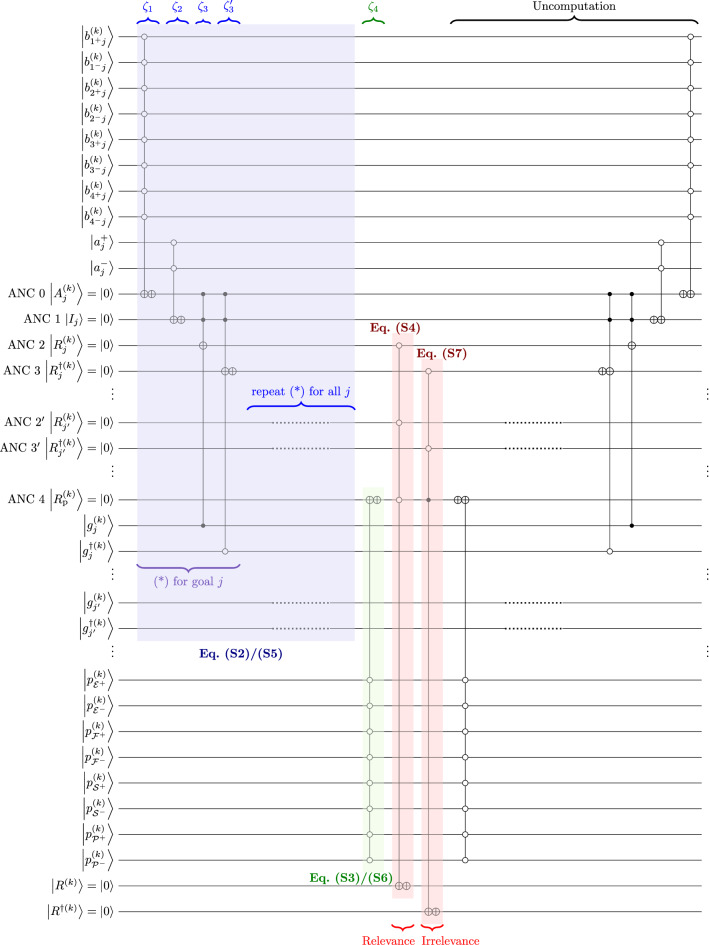
Algorithm for relevance generation. $\zeta _1$, $\zeta _2$, $\zeta _3$ and $\zeta _4$ refer to Eqs. (S2)–(S7).

According to Section S1.2.1, relevance evaluation involves the comparison between encoded features and goals or concerns of the system (e.g., to help the user, to find the charging station). Here, the algorithm for one feature *k* is discussed as an illustration. The qubits involved in this section are listed in Table S2. This process involves a series of beliefs as a bidimensional unipolar scale of an action for feature *k* ($i^{(k)}$) that *facilitates* or *inhibits* goal *j*, denoted as $\bigl|{b^{(k)}_{i^{+}j}} \bigr\rangle$ and $\bigl|{b^{(k)}_{i^{-}j}} \bigr\rangle$, respectively. Here, 4 actions are shown to mimic the 4 general action tendencies as discussed in Section S1.3.3. Goal *j* may be *desired* or *undesired*, represented by $\bigl|{a_j^+} \bigr\rangle$ and $\bigl|{a_j^-} \bigr\rangle$ as a bidimensional unipolar scale. All the abovementioned kets are pre-initialized single qubits representing two-state systems. Every action may affect goal *j*, where the *affect* operator is the disjunction of *facilitates* and *inhibits*. So in Eqs. (S2) and (S5), for a particular $i^{(k)}$ in statement $\zeta _1$, the qubit “any $i^{(k)}$ affects *j*” $\bigl|{A^{(k)}_j} \bigr\rangle$ as an ancilla qubit initialized to be $|{0} \rangle$ is the output of the or operation connected to controls $\bigl|{b^{(k)}_{i^{+}j}} \bigr\rangle$ and $\bigl|{b^{(k)}_{i^{-}j}} \bigr\rangle$ for all $i^{(k)}$ (Fig. 6). Likewise, for statement $\zeta _2$, the ancilla qubit for “goal *j* is *important*” $\bigl|{I_j} \bigr\rangle$ is the disjunction of *desired* and *undesired*, given by the output of the or operation on $\bigl|{a_j^+} \bigr\rangle$ and $\bigl|{a_j^-} \bigr\rangle$ (Fig. 6). Then, the *agree* qubit $\bigl|{g_j^{(k)}} \bigr\rangle$ and the *disagree* qubit $\bigl|{g_j^{\dagger (k)}} \bigr\rangle$ representing statements $\zeta _3$ and $\zeta _3^\dagger$, respectively, are connected in conjunction with $\bigl|{A^{(k)}_j} \bigr\rangle$ and $\bigl|{I_j} \bigr\rangle$ via the and/or operation and produce the ancilla outputs of relevance and irrelevance from goal comparison, $\bigl|{R_j^{(k)}} \bigr\rangle$ and $\bigl|{R_j^{\dagger (k)}} \bigr\rangle$ as bimensional unipolar scales (Fig. 6). The algorithm loops through all goals *j* to produce their respective $\bigl|{R_j^{(k)}} \bigr\rangle$ and $\bigl|{R_j^{\dagger (k)}} \bigr\rangle$.

The determination of relevance from encoded features, i.e., statement $\zeta _4$ in Eqs. (S2) and (S5), is simply an or operation on the perceived weights of all appraisal variables $\bigl|{p^{(k)}_l} \bigr\rangle$, producing the ancilla qubit $\bigl|{R_{\mathrm {p}}^{(k)}} \bigr\rangle$ as a state of a unipolar scale. It is assumed that the negation of it is the irrelevance counterpart. The disjunction of the two kinds of relevance states (for all *j*) gives the final relevance state $\bigl|{R^{(k)}} \bigr\rangle$, and the disjunction of the two kinds of irrelevance states (for all *j*), with one of them as the negated control for $\bigl|{R_{\mathrm {p}}^{(k)}} \bigr\rangle$, gives the final irrelevance state $\bigl|{R^{\dagger (k)}} \bigr\rangle$. After all operations, uncomputation is performed on all ancilla qubits (Fig. 6). Table S2 summarizes the qubits involved in the calculation.

### Valence

**Figure 7:**
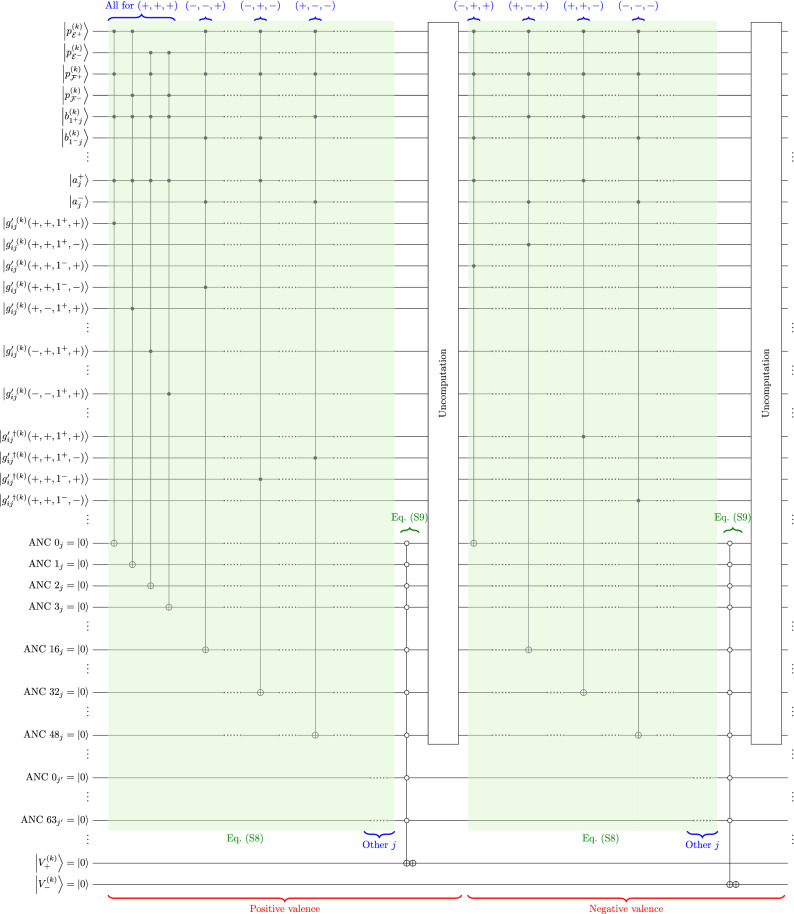
Extract of quantum algorithm for the valence evaluation process. The blue $\left( \cdot ,\cdot ,\cdot \right)$ annotation represents in sequence the indicative ($+$) or counter-indicative (−) variables for *belief* (*facilitate*/*inhibit*), *ambition* (*desired*/*undesired*) and *agreement* (*agree*/*disagree*). All 4 combinations involving $\bigl|{p^{(k)}_{{\mathcal {E}}^{\pm }}} \bigr\rangle$ and $\bigl|{p^{(k)}_{{\mathcal {F}}^{\pm }}} \bigr\rangle$ for $\left( +,+,+\right)$ are depicted as an example. For other $\left( \cdot ,\cdot ,\cdot \right)$ combinations, only the combination with $\bigl|{p^{(k)}_{{\mathcal {E}}^{+}}} \bigr\rangle$ and $\bigl|{p^{(k)}_{{\mathcal {F}}^{+}}} \bigr\rangle$ is shown for conciseness. This figure corresponds to Eqs. (S8) and (S9).

The valence evaluation is based on Eq. (S8) where the kets involved are summarized in Table S2. Positive and negative valences are calculated separately. Whether Eq. (S8) gives positive or negative valence depends on the qubits chosen in statements $\zeta '_3$ ($\bigl|{b^{(k)}_{i^\pm j}} \bigr\rangle$, altogether no. of actions $\times 2$ qubits), $\zeta '_4$ ($\bigl|{a_j^\pm } \bigr\rangle$) and $\zeta '_5$. In $\zeta '_5$, an *agree* qubit $\bigl|{g'{}^{(k)}_{\!ij}} \bigr\rangle$ and a *disagree* qubit $\bigl|{g'{}^{\dagger (k)}_{\!ij}} \bigr\rangle$ represent the bidimensional unipolar agreement scale for one combination of qubits chosen in $\zeta '_1$ to $\zeta '_4$. The kets of *agree* are specified as $\bigl|{g'{}^{(k)}_{\!ij}\!\bigl( \pm _{\mathcal {E}},\pm _{\mathcal {F}},i^\pm ,\pm _{a_j}\bigr) } \bigr\rangle$, which is associated to the combination $\bigl|{p^{(k)}_{\mathcal {E}^\pm }} \bigr\rangle$, $\bigl|{p^{(k)}_{\mathcal {F}^\pm }} \bigr\rangle$, $\bigl|{b^{(k)}_{i^{\pm }j}} \bigr\rangle$ and $\bigl|{a^{\pm }_j} \bigr\rangle$ (Fig. 7), so are the kets of *disagree*. Therefore, with 4 possible actions for feature *k*, there are 64 *agree* qubits and 64 *disagree* qubits per goal per feature on the agreement of $\left( \zeta '_1 \wedge \zeta '_2 \wedge \zeta '_3 \wedge \zeta '_4\right)$ for the respective combinations of qubits. One ancilla qubit per combination is required to store the intermediate result of Eq. (S8), which is the output of the generalized Toffoli and gate connected to the respective controls of qubits for $\zeta '_1$ to $\zeta '_4$. The operation of Eq. (S8) loops through all goals *j*. The disjunction of the ancilla qubits (for all goals *j*) produces the final valence [Eq. (S9)], followed by the uncomputation of the ancilla qubits. Calculating positive and negative valence ($\bigl|{V^{(k)}_+} \bigr\rangle$ and $\bigl|{V^{(k)}_-} \bigr\rangle$) separately, 64 ancilla qubits per goal are required in this scenario of the process.

Whether the collection of selected qubits generates an output for positive or negative valence depends on qubits involved in $\zeta '_3$, $\zeta '_4$, and $\zeta '_5$. According to Eq. (S8), a rule of positive valence is generated from a collection of both indicative ($+$) or both counter-indicative (−) qubits for $\zeta '_3$ and $\zeta '_4$ together with the *agree* qubit in $\zeta '_5$, or that only one of the qubits in $\zeta '_3$ and $\zeta '_4$ is indicative, combined with a *disagree* qubit, and vice versa. Figure 7 shows part of the quantum circuit as an illustration.

### Use intentions

**Figure 8:**
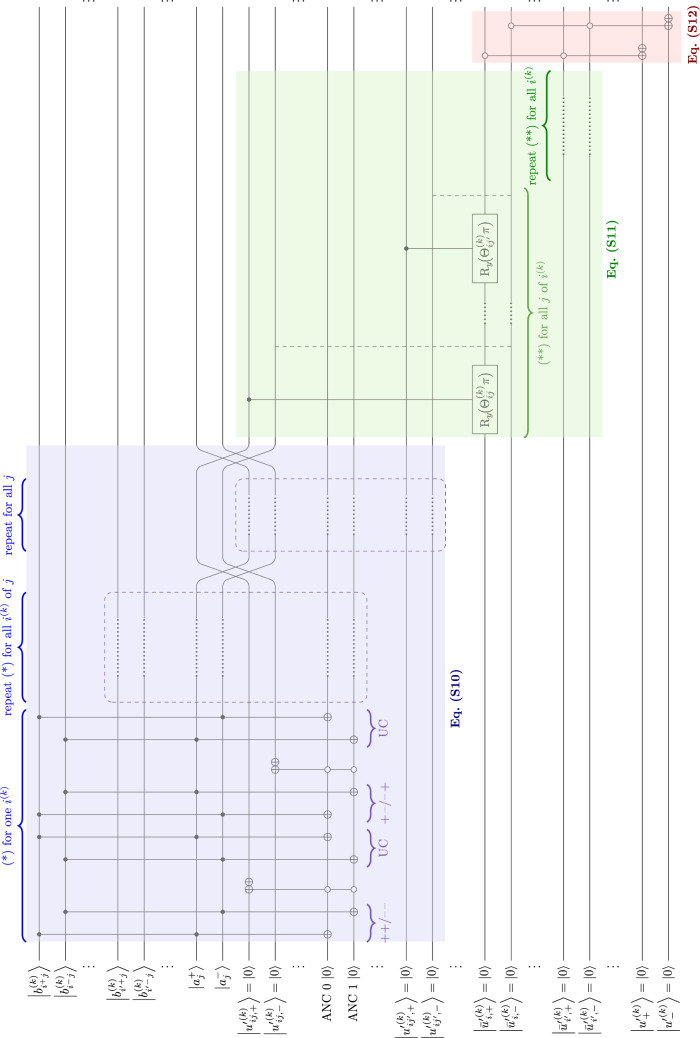
Extract of quantum algorithm for the utility evaluation process. UC: uncomputation. For simplicity, $\Theta ^{(k)}\!\bigl (u'{}^{(k)}_{\!ij}\bigr ) = \Theta ^{(k)}_{ij}$. The annotation $\mu \nu = ++,--,+-,-+$ represents the combination of $\bigl|{b^{(k)}_{i^\mu j}} \bigr\rangle$ and $\bigl|{a_j^{\nu }} \bigr\rangle$.

According to Section S1.3.1, the utility of an action as the intermediate variable is calculated first. The qubits involved in this section are listed in Table S3. The expected utility $u'^{(k)}_{\!ij}$ defined in Eq. (S10) is modeled as an indicative $\bigl|{u'^{(k)}_{\!ij,+}} \bigr\rangle$ and a counter-indicative $\bigl|{u'{}^{(k)}_{\!ij,-}} \bigr\rangle$ qubit. Positive and negative values of $u'^{(k)}_{\!ij}$ are associated with $\bigl|{u'{}^{(k)}_{\!ij,+}} \bigr\rangle$ and $\bigl|{u'{}^{(k)}_{\!ij,-}} \bigr\rangle$, respectively. The multiplication in Eq. (S10) is interpreted as the Toffoli gate. Then, $\bigl|{u'{}^{(k)}_{\!ij,+}} \bigr\rangle$ is toggled with the conjunction of $\bigl|{b_{i^{+}j}^{(k)}} \bigr\rangle$ and $\bigl|{a_j^{+}} \bigr\rangle$ or that of $\bigl|{b_{i^{-}j}^{(k)}} \bigr\rangle$ and $\bigl|{a_j^{-}} \bigr\rangle$, and $\bigl|{u'{}^{(k)}_{\!ij,-}} \bigr\rangle$ with the conjunction of $\bigl|{b_{i^{+}j}^{(k)}} \bigr\rangle$ and $\bigl|{a_j^{-}} \bigr\rangle$ or $\bigl|{b_{i^{-}j}^{(k)}} \bigr\rangle$ and $\bigl|{a_j^{+}} \bigr\rangle$, as shown in Fig. 8. The two toggling conditions are fed into two ancilla qubits, using the Toffoli gates, which are then connected to the expected utility qubits using the or operation, followed by uncomputation. The above operations are performed on all actions $i^{(k)}$ per goal for a particular feature *k*.

For each (counter-)indicative expected utility, the mean expected utility over goals $\bigl|{\bar{u}'{}^{(k)}_{\!i,\pm }} \bigr\rangle$ may be calculated with a weight $\Theta _i^{(k)}\!\bigl (u'{}^{(k)}_{\!ij}\bigr )$ [Eq. (S11)]. Although there is no simple quantum circuit that can mimic exactly  Eq. (S11), by interpreting the mean as an entity composed of different factors, the mean value can be modeled as a superposition of the states of these factors, each of which could rotate $\bigl|{\bar{u}'{}^{(k)}_{\!i,\pm }} \bigr\rangle$ in the Bloch-sphere at some angle. Assuming $\sum _j \Theta _i^{(k)}\!\bigl (u'{}_{\!ij}^{(k)}\bigr ) = 1$, i.e., a maximum possible rotation of $\pi$,  Eq. (S11) can be translated into a series of $\hbox {R}_y$ rotations with an angle of $\Theta _i^{(k)}\!\bigl (u'{}_{\!ij}^{(k)}\bigr ) \pi$, depending on the goal *j* for every $\bigl|{\bar{u}'{}^{(k)}_{\!i,\pm }} \bigr\rangle$. Such formalism resembles Eq. (S11) satisfactorily (Fig. S1). The indicative and counter-indicative utilities in Eq. (S12) are then modeled as a pair of qubits ($\bigl|{u'{}^{(k)}_{\!\pm }} \bigr\rangle$), given as the output of the or operations with the control qubits of $\bigl|{\bar{u}'{}^{(k)}_{\!i,\pm }} \bigr\rangle$ corresponding to all of the respective $i^{(k)}$ (Fig. 8).

**Figure 9:**
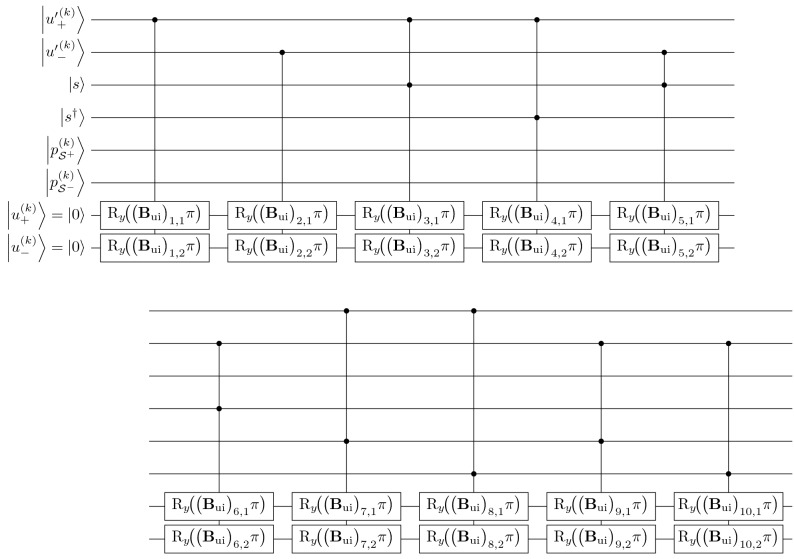
Extract of quantum algorithm for the evaluation process of use intentions, corresponding to Eqs. (S13) and (S14).

Finally, use intentions [Eq. (S13)] as a composition of different components as depicted in the weight matrix ${\mathbf {B}}_{\mathrm {ui}}$ in Eq. (S14) may be modeled as a series of $\hbox {R}_y$ rotations, similar to the mean expected utility. Since $\vec {r}_{\text {ui}}^{(k)} \in {\mathbb {R}}^{10}$, the indicative and counter-indicative qubits of use intentions $\bigl|{u^{(k)}_\pm } \bigr\rangle$ are respectively composed of 10 conditional $\hbox {R}_y$ rotations, under the angles of the product of the corresponding coefficient $\left( {\mathbf {B}}_{\mathrm {ui}}\right) _{\mu \nu }$ and $\mathrm {\pi }$ (Fig. 9).

### Involvement–distance trade-off

**Figure 10:**
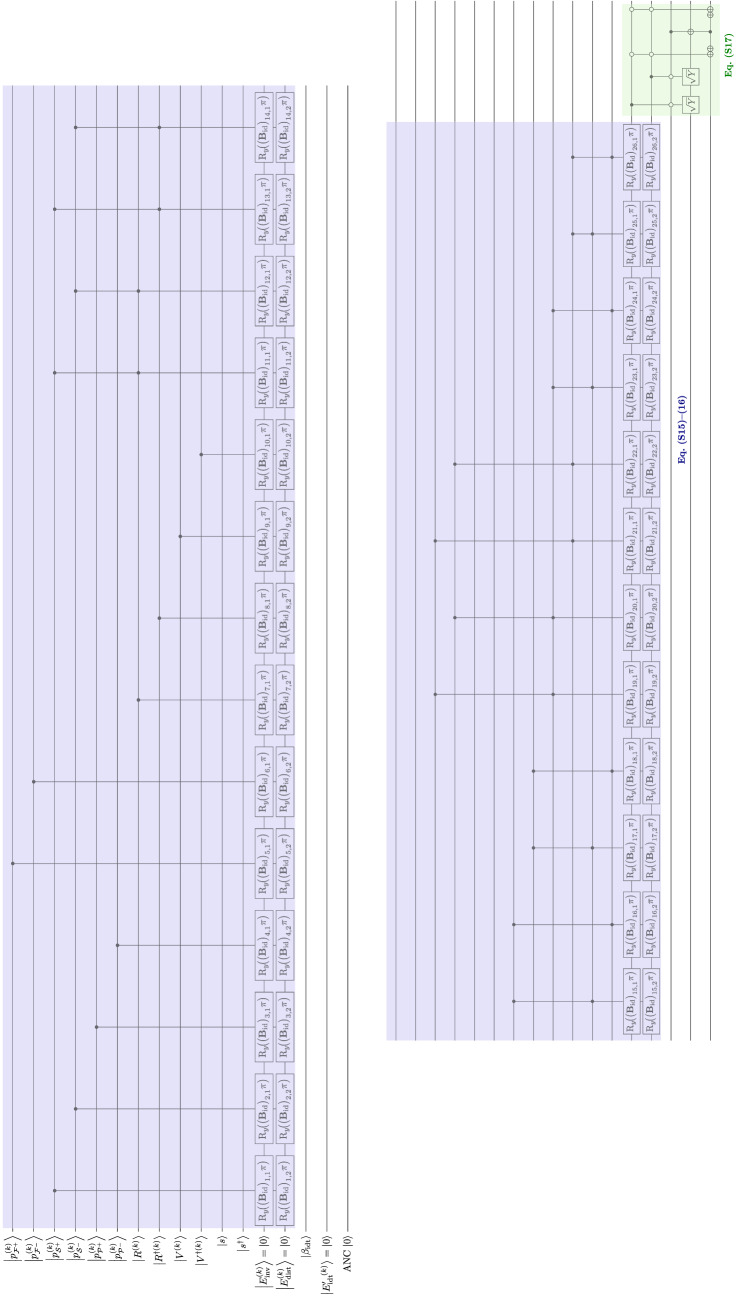
Extract of quantum algorithm for the involvement–distance trade-off.

According to Eqs. (S15) and (S16), involvement and distance, collectively referred to as engagement, comprise various factors superposing together to give a collective (superposed) state, similar to use intentions. To calculate the involvement $\bigl|{E^{(k)}_{\mathrm {inv}}} \bigr\rangle$ and distance $\bigl|{E^{(k)}_{\mathrm {dist}}} \bigr\rangle$ qubits as denoted in Table S4, a similar quantum algorithm is implemented of conditional $\hbox {R}_y$ rotations of angles determined by the weight matrix elements (Fig. 10). The involvement–distance trade-off qubit $\bigl|{E{{}'}_{\!\text {idt}}^{(k)}} \bigr\rangle$, according to Eq. (S17), may be modeled by introducing the compensation factor qubit for involvement and distance $|{\beta _{\text {idt}}} \rangle$. $\bigl|{E{{}'}_{\!\text {idt}}^{(k)}} \bigr\rangle$ undergoes two $\hbox {R}_y$ rotations due to the latter term of an angle of maximum of $\mathrm {\pi }/2$ weighted by $1-\beta _{\mathrm {idt}}$, which can be constructed by the $\sqrt{Y}$ gate connected to $|{\beta _{\text {idt}}} \rangle$ as the $|{0} \rangle$-triggered control and $\bigl|{E^{(k)}_{\mathrm {inv}}} \bigr\rangle$ or $\bigl|{E^{(k)}_{\mathrm {dist}}} \bigr\rangle$ as the $|{1} \rangle$-triggered control. The former term is achieved by a $|{\beta _{\text {idt}}} \rangle$-conditioned or operation with $\bigl|{E^{(k)}_{\mathrm {inv}}} \bigr\rangle$ and $\bigl|{E^{(k)}_{\mathrm {dist}}} \bigr\rangle$ as the controls (Fig. 10). The ancilla qubit is uncomputed after use. The square root gate characterizes the division of 2. As revealed in Eq. (S17), the two terms represent different operations for compensation^1^. In the quantum circuit, the or operation and the rotation indicate the two operations. The mix of the schemes (trade-off) is accomplished by employing the entanglement formalism using the qubit $|{\beta _{\text {idt}}} \rangle$. In terms of mathematics, let $\theta _{\mathrm {i}}$, $\theta _{\mathrm {d}}$ and $\theta _{\mathrm {\beta }}$ denote the polar angles of the states $\bigl|{E^{(k)}_{\mathrm {inv}}} \bigr\rangle$, $\bigl|{E^{(k)}_{\mathrm {dist}}} \bigr\rangle$ and $|{\beta _{\text {idt}}} \rangle$ in the Bloch-sphere representation as in Eq. (1). Then the probability of $\bigl|{E'{}_{\!\text {idt}}^{(k)}} \bigr\rangle = |{1} \rangle$ under measurement is

6 $$\begin{aligned} \Bigl|{{\bigl\langle }{E{{}'}_{\!\text {idt}}^{(k)}}\bigm|{1}{\bigr\rangle }} \Bigr|^2 = \sin ^2\frac{\theta _{\mathrm {\beta }}}{2}\left( \sin ^2\frac{\theta _{\mathrm {d}}}{2} + \sin ^2\frac{\theta _{\mathrm {i}}}{2} - \sin ^2\frac{\theta _{\mathrm {d}}}{2} \sin ^2\frac{\theta _{\mathrm {i}}}{2} \right) + \frac{1}{2} \cos ^2\frac{\theta _{\mathrm {\beta }}}{2}\left( \sin ^2\frac{\theta _{\mathrm {d}}}{2} + \sin ^2\frac{\theta _{\mathrm {i}}}{2} \right) \end{aligned}$$

where the first term corresponds to the or operation and the second to the successive $\hbox {R}_y$ rotations. This is analogous to Eq. (S17), justifying the circuit design.

### Satisfaction

**Figure 11:**
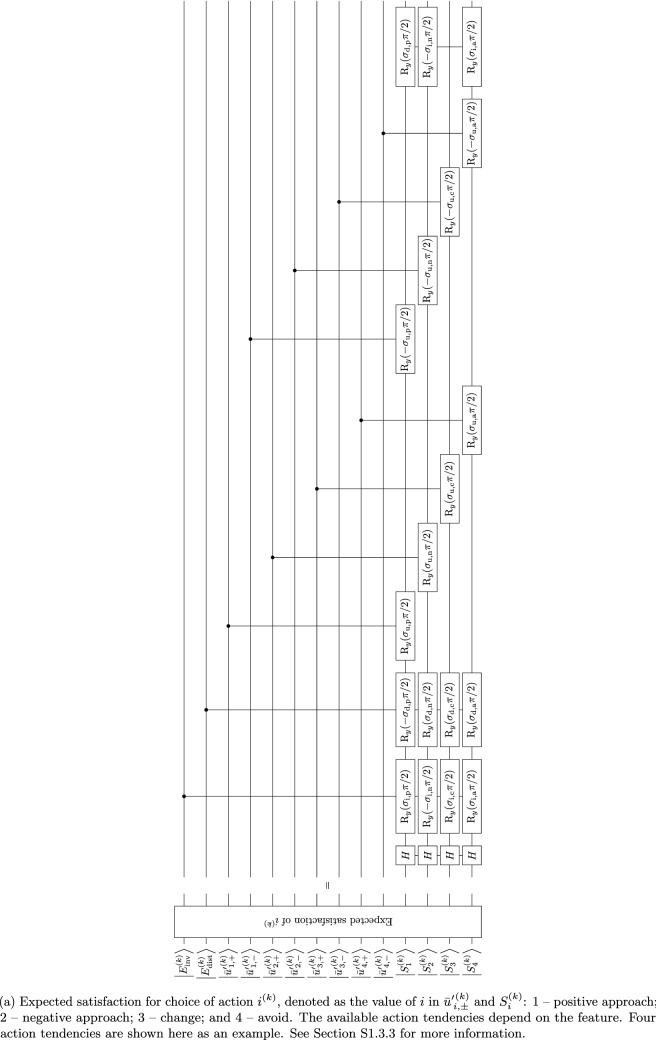

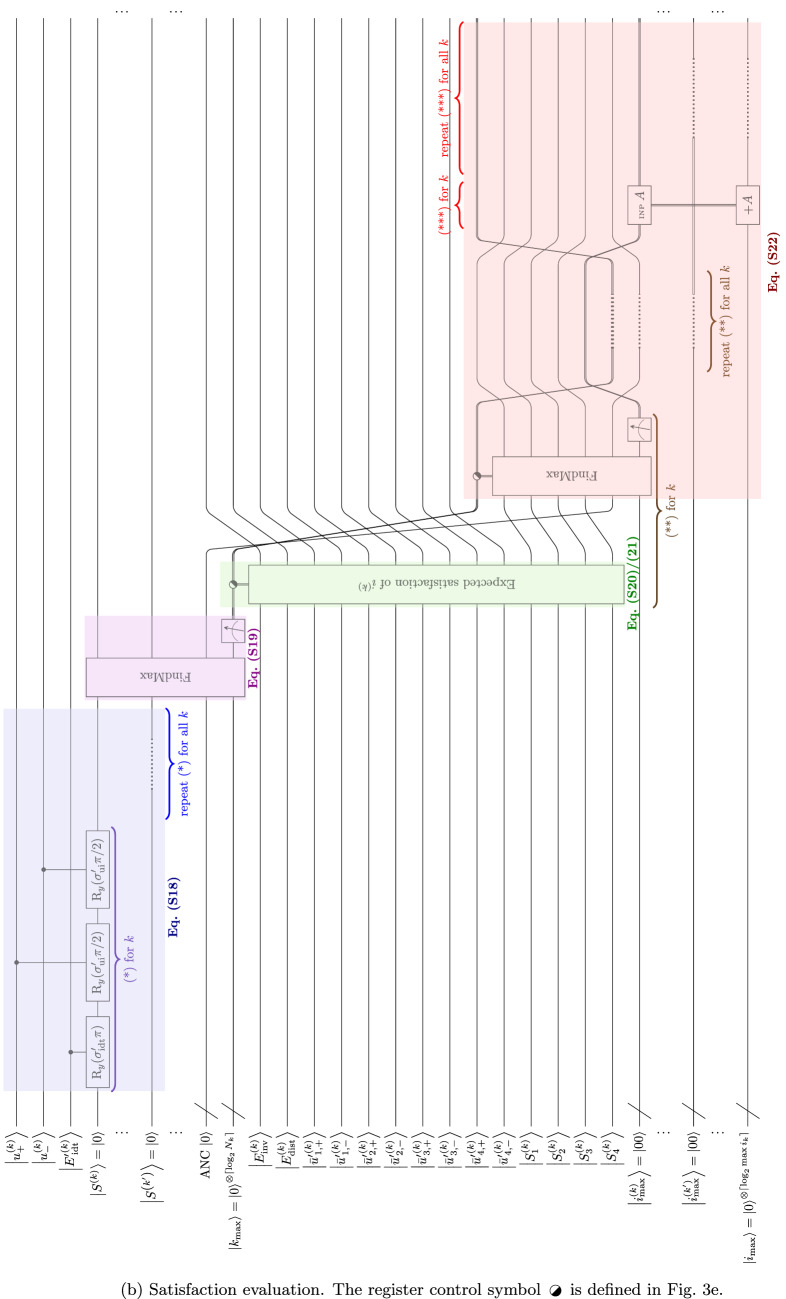
Extract quantum algorithm for satisfaction evaluation and decision-making (choice of action).

Satisfaction evaluation starts with calculating the expected satisfaction [Eq. (S18)], which is the $\bigl|{S^{(k)}} \bigr\rangle$ qubit in the quantum algorithm (Table S5). Considering the satisfaction value as a combined state of the involvement–distance trade-off and use intentions, it is modeled as three successive $\hbox {R}_y$ rotations conditional to $\bigl|{u^{(k)}_+} \bigr\rangle$, $\bigl|{u^{(k)}_-} \bigr\rangle$ and $\bigl|E'{}^{(k)}_{\!\mathrm {idt}} \bigr\rangle$ with angles of their respective weights ($\sigma '_{\mathrm {idt}}$ and $\sigma '_{\mathrm {ui}}/2$) of $\mathrm {\pi }$. This operates on all *k* (Fig. 11b). Then, the maximum of $\bigl|{S^{(k)}} \bigr\rangle$ for different *k* is found using the maximum search algorithm in Section “Circuit components for quantum logic”. After the measurement, a definite integer is stored in a classical register, denoting the value of $k_{\max }$ [Eq. (S19)]. The value of $k_{\max }$ corresponds to the feature that is observed (selected) for the greatest satisfaction and allows for the observed action (see Section S1.3.3). The register then is connected to the evaluation block for the expected satisfaction for action $i^{(k)}$ ($\bigl|{S_i^{(k)}} \bigr\rangle$) for each *k* as the control. The control corresponds to the value of *k* (see Fig. 3e). The evaluation block for satisfaction value for action $i^{(k)}$ is the weighted sum of $E_{\text {inv}}^{(k_{\max })}$, $E_{\text {dist}}^{(k_{\max })}$, and $\bar{u}'{}^{(k_{\max })}_{\!i}$ (Eqs. (S20) and (S21)]. For ease of comparison, $S^{(k)}_i \in [-1,1]$ is regarded as a unidimensional unipolar scale as if we transform the range to [0, 1]. Then $\bigl|{S^{(k)}_i} \bigr\rangle = \frac{|{0} \rangle +|{1} \rangle }{2}$ is the neutral point of the scale as the initial state, given by the Hadamard gates (Fig. 11a). It is followed by successive $\hbox {R}_y$ rotations with the factors of qubits $\bigl|{E_{\text {inv}}^{(k)}} \bigr\rangle$, $\bigl|{E_{\text {dist}}^{(k)}} \bigr\rangle$, $\bigl|{\bar{u}'{}_{\!i,+}^{(k)}} \bigr\rangle$ and $\bigl|{\bar{u}'{}_{\!i,-}^{(k)}} \bigr\rangle$ as the controls and the angles of the respective fractions of $\mathrm {\pi }/2$. Since $\bar{u}'{}^{(k_{\max })}_{\!i} \in [-1,1]$ indicating that $\bar{u}'{}^{(k_{\max })}_{\!i}$ could contribute negatively to $S^{(k)}_i$, $\bigl|{\bar{u}'{}_{\!i,+}^{(k)}} \bigr\rangle$ and $\bigl|{\bar{u}'{}_{\!i,-}^{(k)}} \bigr\rangle$ would be controls for positive and negative angles of $\hbox {R}_y$ rotations, respectively. Note that the algorithm is present on the qubits for every *k*, but due to the classical nature of the $k_{\max }$ register, only the evaluation block associated with $k_{\max }$ runs eventually. Then, retaining the *k* register as the control, the $\bigl|{S_i^{(k)}} \bigr\rangle$ qubits for all $i^{(k)}$ are fed to the maximum search algorithm for each *k* to find $i_{\max }$, similar to the case for determining $k_{\max }$ [Eq. (S22)]. Measurement is performed on the resultant register for $i_{\max }$ to get a particular $i_{\max }$ value. Again, only the block associated with $k_{\max }$ would be implemented in practice. Since every *k* has an evaluation block containing an $i_{\max }$ register, another register is needed to store the final output of $i_{\max }$ among all *k*. This register adds up the value of the $i_{\max }$ calculated in all *k*. Because only one of the evaluation blocks among different *k* runs, the value of $i_{\max }$ is 0 for all *k* except the one of $k_{\max }$. Therefore, the resultant value of the register equals $i_{\max }$ of $k_{\max }$. Since $i_{\max }$ and $k_{\max }$ are found, the action for the dominant feature is determined. The affective decision-making process is complete.

## Discussion

Section “The Quantum Coppélia model” shows that Q-Coppélia is an elaborate system and reveals that there will be technical difficulties in doing the actual simulations, albeit for the extensive number of qubits needed for its implementation. However, as a theoretical exercise, it is important to know how much quantum-computing power would be needed. Moreover, theoretically, we want to see what our quantum logics can do more or different from fuzzy approaches. Therefore, in this section, a conceptual comparison is made between the fuzzy-based Silicon Coppélia system and the quantum-based Q-Coppélia system.

### Vagueness and ambiguity

Silicon Coppélia employs fuzzy logic to model the vagueness of the agency’s affective state. Fuzzy logic fuzzifies the ordinary 0 and 1 using membership functions bearing a continuous value [0, 1]. Instead of a discrete interpretation of True or False, e.g., He is/is not *good*, fuzzy logic interprets this as having a certain degree of *good*. This means that the verdict is neither exactly *good* nor entirely not *good*, but perhaps “somewhat” *good*. This “somewhat” differentiates a fuzzy set from a crisp set, but note that this “somewhat” is not typically interpreted as a “mix” of both yes and no, but as being “uncertain” of whether it is yes or no. The extent to which a feature belongs to a particular set depends on the value of the membership function, i.e.,  it is the value itself that determines the state. In Silicon Coppélia, the decision is made by converting these membership functions into indicators such as use intentions, involvement, distance, and eventually satisfaction, followed by comparing the satisfaction values that are associated with different features and action tendencies.

**Figure 12:**
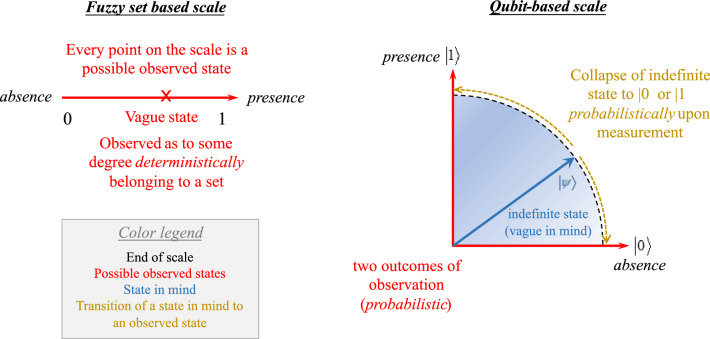
Comparison of vagueness in terms of fuzzy-set–based and qubit-based scales. The text color denotes the type of description for ease of comparison. The concepts associated with the blue and orange texts only appear in quantum logic, and thus are absent in the fuzzy-set–based scale.

Q-Coppélia has a slightly different interpretation of vagueness. The appraisal variables are modeled as two-state systems of states $|{0} \rangle$ and $|{1} \rangle$ (corresponding to, for example, *not good* and *good*, respectively) for which superposition is allowed. A pure state as a *composite of the two states* is constructed from their linear combination, with their amplitudes indicating the probabilities, as shown in the Bloch sphere (Fig. 2e). The vagueness comes from the inexactness of the state with respect to the basis of measurement (computational basis) until a measurement (observation) is performed. More importantly, the kets $|{0} \rangle$ and $|{1} \rangle$ are orthonormal. Therefore, the quantum two-state representation of vagueness is indicated by a vector of unit length in the Hilbert space composed of *two* basis vectors as the *outcome*. It is qualitatively different from the physical significance of the ends of a fuzzy scale, which indicate only the extremes of membership of complementary contexts, i.e., A and not A. Whereas the value of the membership function in fuzzy logic defines the degree to which a state is in a particular set (fuzziness), *deterministically* using a *continuous* linear scale, quantum logic distinguishes the state itself and the state after a measurement where the numbers (probability amplitudes) indicate the probabilities of getting the possible outcome. Such measurement is regarded as a process of decision-making. The measurement returns either state in the computational basis ($|{0} \rangle$ or $|{1} \rangle$) based on the probability distribution. In other words, the ket denotes vagueness by indicating the probability of having the state either $|{0} \rangle$ or $|{1} \rangle$ instead of the degree to which a state belongs to the set. If the measurement is associated with the observable $${\hat{A}} = \begin{bmatrix} 0 &{} 0 \\ 0 &{} 1 \end{bmatrix}$$ as discussed in Section “Encoding features”, then the expected value would be the same as the value of the membership function. In particular, the state that can be observed is a probabilistic outcome of either “extreme”, and the ensemble under repeated measurements *converges* to the value of the membership function. Fig. 12 illustrates the comparison of vagueness in terms of fuzzy-set–based and qubit-based scales.

Whereas vagueness is represented by fuzzified quantities, ambiguity is indicated by the so-called multi-valued quantities. In Silicon Coppélia, bidimensional unipolar scales are employed so that an appraisal variable, such as aesthetics in Section 12.4^1^, can possess multiple values (in the set of “beautiful” and in the set of “ugly”). Ambiguity is accomplished by setting both poles at non-zero for evaluation. In Q-Coppélia, ambiguity is not only revealed through the use of bidimensional unipolar scales but also naturally, as the quantum properties of mixed states. As a statistical mixture of pure states, if a pure state of the qubit of *beautiful* denotes a particular perspective towards aesthetics, then a mixed state is a collection of different perspectives (e.g., so ugly that it becomes beautiful again). In this sense, ambiguity manifests as it is uncertain which perspective the agency holds. We consider the capability of having multiple perspectives for even one of the bidimensional unipolar scales to be an extension of the Silicon Coppélia approach. It should be noted that, although both bidimensional unipolar scales and mixed states facilitate an ambiguous state, the use of bidimensional unipolar scales allows for a different account of evaluation because distinct weight factors may be assigned to individual scales (e.g., Eqs. (S14) and (S16)). Indeed, humans may treat positive and negative aspects of an appraisal differently, and thus, our quantum representation can satisfy such general psychological considerations.

Section “Circuit components for quantum logic” introduced the rotation formalism and the entanglement formalism for generating a purity-preserved state of vagueness and a mixed state of ambiguity, respectively. Operations of the two formalisms may be subject to extrinsic conditions, which are imposed through additional control qubits. The use of controlled operations leads to mixed states, in the vein of the entanglement formalism. Nonetheless, it should be noted that the resulting states of the two controlled formalisms possess distinct characteristics as the degree of mixing of the rotation formalism is generally less than the entanglement formalism, implying a psychological state of less ambiguity. To illustrate this, we first recall the density matrices of the states generated from the rotation formalism and the entanglement formalism without additional controls. Assuming a weight $\alpha$, the density matrix under the rotation formalism is

7 $$\begin{aligned} {\hat{\rho }}_{\mathrm {R}_y}&= \mathrm {R}_y \left( \alpha \mathrm {\pi }\right) |{0} \rangle \langle {0} |\ \mathrm {R}_y \left( \alpha \mathrm {\pi }\right) ^\dagger \nonumber \\&= \begin{bmatrix} \cos \frac{\alpha \mathrm {\pi }}{2} &{} -\sin \frac{\alpha \mathrm {\pi }}{2} \\ \sin \frac{\alpha \mathrm {\pi }}{2} &{} \cos \frac{\alpha \mathrm {\pi }}{2} \end{bmatrix} \begin{bmatrix} 1 \\ 0 \end{bmatrix} \begin{bmatrix} 1&0 \end{bmatrix} \begin{bmatrix} \cos \frac{\alpha \mathrm {\pi }}{2} &{} \sin \frac{\alpha \mathrm {\pi }}{2} \\ -\sin \frac{\alpha \mathrm {\pi }}{2} &{} \cos \frac{\alpha \mathrm {\pi }}{2} \end{bmatrix} \nonumber \\&= \begin{bmatrix} \frac{1}{2}\left( 1+\cos \alpha \mathrm {\pi }\right) &{} -\frac{1}{2}\sin \alpha \mathrm {\pi } \\ \frac{1}{2}\sin \alpha \mathrm {\pi } &{} \frac{1}{2}\left( 1-\cos \alpha \mathrm {\pi }\right) \end{bmatrix} \end{aligned}$$

i.e., the quantum state is $|{\psi } \rangle = \cos \frac{\alpha \mathrm {\pi }}{2} |{0} \rangle + \sin \frac{\alpha \mathrm {\pi }}{2} |{1} \rangle$, which is a pure state (${{\,\mathrm{Tr}\,}}\!\bigl ({\hat{\rho }}_{\mathrm {R}_y}^2\bigr ) = 1$). In contrast, the density matrix under the entanglemet formalism with $|{\alpha } \rangle = \cos \frac{\alpha \mathrm {\pi }}{2} |{0} \rangle + \sin \frac{\alpha \mathrm {\pi }}{2} |{1} \rangle$ is

8 $$\begin{aligned} {\hat{\rho }}_{\mathrm {CNOT}}&= \big [\mathrm {CNOT}\left( |{0} \rangle \otimes |{\alpha } \rangle \right) \big ] \big [\mathrm {CNOT}\left( |{0} \rangle \otimes |{\alpha } \rangle \right) \big ]^\dagger \nonumber \\&= \left[ |{0} \rangle \otimes \cos \frac{\alpha \mathrm {\pi }}{2} |{0} \rangle + X|{0} \rangle \otimes \sin \frac{\alpha \mathrm {\pi }}{2}|{1} \rangle \right] \left[ |{0} \rangle \otimes \cos \frac{\alpha \mathrm {\pi }}{2} |{0} \rangle + X|{0} \rangle \otimes \sin \frac{\alpha \mathrm {\pi }}{2}|{1} \rangle \right] ^\dagger \nonumber \\&= \left[ \cos \frac{\alpha \mathrm {\pi }}{2} |{0} \rangle \otimes |{0} \rangle + \sin \frac{\alpha \mathrm {\pi }}{2} |{1} \rangle \otimes |{1} \rangle \right] \left[ \cos \frac{\alpha \mathrm {\pi }}{2} |{0} \rangle \otimes |{0} \rangle + \sin \frac{\alpha \mathrm {\pi }}{2} |{1} \rangle \otimes |{1} \rangle \right] ^\dagger \nonumber \\&= \begin{bmatrix} \frac{1}{2}\left( 1+\cos \alpha \mathrm {\pi }\right) &{} 0 &{} 0 &{} \frac{1}{2}\sin \alpha \mathrm {\pi } \\ 0 &{} 0 &{} 0 &{} 0 \\ 0 &{} 0 &{} 0 &{} 0 \\ \frac{1}{2}\sin \alpha \mathrm {\pi } &{} 0 &{} 0 &{} \frac{1}{2}\left( 1-\cos \alpha \mathrm {\pi }\right) \end{bmatrix} \end{aligned}$$

where the partial trace gives the density matrix of the target qubit as

9 $$\begin{aligned} {\hat{\rho }}_1 = \begin{bmatrix} \frac{1}{2}\left( 1+\cos \alpha \mathrm {\pi }\right) &{} 0 \\ 0 &{} \frac{1}{2}\left( 1-\cos \alpha \mathrm {\pi }\right) \end{bmatrix} \end{aligned}$$

with a purity of $\gamma = {{\,\mathrm{Tr}\,}}\!\left( {\hat{\rho }}_1^2\right) = \frac{1}{2}\left( 1+\cos ^2\alpha \mathrm {\pi }\right)$, indicating a mixed state, of which the ambiguity depends on $|{\alpha } \rangle$. Such a state comprises of $|{0} \rangle \langle {0} |$ and $|{1} \rangle \langle {1} |$ only, and is different from that in Eq. (7) in a sense that the absence of the off-diagonal elements in Eq. (9) indicates a state of either $|{0} \rangle$ or $|{1} \rangle$ as a statistical mixture without interference. The case with an additional control qubit is similar. Figure S2 shows the results of the purity of the target state under a controlled rotation formalism and a controlled entanglement formalism with one extrinsic condition (control qubit). The blue region represents a fully mixed state ($\gamma = 0.5$). For the rotation formalism (Fig. S2a), a pure state can be retained except when the control qubit possesses either a state around the equator in the Bloch sphere or a fully mixed state with an angle of rotation approaching $\mathrm {\pi }$, resembling a Bell state. For other angles of rotation, the component undergoing the $\hbox {R}_y$ rotation results in a state that does not align with $|{1} \rangle$ in the Bloch sphere, reducing its mixed property. In contrast, the symmetric circuit structure of the weight and condition qubits (as the control qubits) for the entanglement formalism (Fig. S2b) gives a mixed state when either of them possesses either a state around the equator in the Bloch sphere or a fully mixed state with an angle of rotation approaching $\mathrm {\pi }$, or more generally, $\left( \mathrm {\pi } - \theta _0\right) ^2 + \left( \mathrm {\pi } - \theta _1\right) ^2 = \left( \frac{\mathrm {\pi }}{2}\right) ^2$. That way, a larger blue area results for the entanglement formalism, i.e., the entanglement formalism leads to more mixed states in general.

### Information feedback and parallel processing

In Silicon Coppélia, the whole appraisal process is essentially a mapping of values, i.e., the equations in Section S1 take up some variables as the input (domain of the function) and return some other variables as the output (range of the function). The values of the input variables remain unchanged after the process. In Q-Coppélia, however, the so-called input variables may change after a process. This is usually through quantum entanglement using controlled operations. The control qubits are often treated as a condition to trigger the target, and therefore regarded as the active input of a process. Interestingly, a controlled $\hbox {R}_y$ operation as depicted in Eq. (3) could alter the state of the control qubit after the rotation.

Suppose a control qubit with the state $|{\psi _0} \rangle = \cos \frac{\theta _0}{2} |{0} \rangle + \sin \frac{\theta _0}{2} |{1} \rangle$ is connected to a target qubit initially in $|{\psi _1} \rangle = |{0} \rangle$ via an $\hbox {R}_y$ gate with an angle of rotation $\theta _1$. After the operation, the density matrix of the control qubit becomes

10 $$\begin{aligned} \bigl|{\psi '_0} \bigr\rangle \bigl\langle {\psi '_0} \bigr| = \begin{bmatrix} \cos ^2\frac{\theta _0}{2} &{} \frac{1}{2}\cos \frac{\theta _1}{2}\sin {\theta _0} \\ \frac{1}{2}\cos \frac{\theta _1}{2}\sin {\theta _0} &{} \sin ^2\frac{\theta _0}{2} \end{bmatrix} \end{aligned}$$

which is different from the initial state

11 $$\begin{aligned} \bigl|{\psi _0} \bigr\rangle \bigl\langle {\psi _0} \bigr| = \begin{bmatrix} \cos ^2\frac{\theta _0}{2} &{} \frac{1}{2}\sin {\theta _0} \\ \frac{1}{2}\sin {\theta _0} &{} \sin ^2\frac{\theta _0}{2} \end{bmatrix}. \end{aligned}$$

in the sense that $\theta _1$ is now involved. Whereas the probability distribution of obtaining $|{0} \rangle$ and $|{1} \rangle$ remains unchanged because the diagonal elements are the same, the purity of the control qubit becomes $1-\frac{1}{2}\sin ^2\theta _0 \sin ^2\frac{\theta _1}{2}$ after the rotation, i.e., the control qubit becomes a mixed state. The purity decreases with $\theta _1$, implying that the state of the control becomes more ambiguous with a larger $\theta _1$. To understand its physical significance, that of a rotation should be explained first. A rotation can be interpreted as switching one perspective (belief) to another. If the state is switched (rotated) from one end (e.g., $|{0} \rangle$) to the other end (e.g., $|{1} \rangle$), the original belief is replaced by an opposite one, similar to negation in fuzzy logics^33^. In other words, the evidence of transforming the belief is strong. Because an indefinite state may be represented by superposition, which is a rotation within the Bloch sphere, a rotation of some angle may be understood as a replacement of belief by another rotation of weaker evidence. Given $\theta _1 = \alpha \mathrm {\pi }$, $\alpha$ as the weight factor may indicate the strength of the evidence for the transformation.

The generation of the mixed state of the control qubit after the rotation implies that the perspective of the target can influence that which the control qubit possesses. A belief transformation not only alters the perspective of the target but also affects all states entangled in it. It is analogous to a feedback loop (e.g., in a feedback amplifier) where the information of the output is correlated to the input *without causal effects*. When the target belief is transformed due to the “control” belief, the original control belief is also affected by the transformed belief in a way that an additional perspective is involved due to entanglement, resulting in ambiguity. The stronger the evidence for the belief transformation, the greater the value of $\theta _1$ and the stronger the perspective contributing to the control belief, resulting in greater ambiguity (a more mixed state). The entanglement property facilitates parallel processing of cognitive functions, carrying out many operations concurrently. It is qualitatively different from mere serial processing, where only one operation can be executed at a time^34^. Silicon Coppélia is mostly executed as a serial process (except that the expected satisfaction evaluation for different features may be optionally processed in parallel)^31^. Quantum entanglement naturally processes correlations between different variables in one operation, resembling parallel processing. The “feedback” does not require classical, possibly computationally intensive algorithms such as recurrence relations, iteration for convergence, or explicitly resolving the general relation under equilibrium (optimization), which is merit for high-performance and efficient affective computation. The connectionist networks and embodied approaches as collective intelligences emphasize the need for distributed parallel processing to mimic the capacity for contextual responses of cognitive processing in the brain^35^. A distributed network resembles information processing as patterns of activation in a neural network across various layers. This coincides with the nature of quantum processes in two aspects: (1) information is regarded as patterns of qubits processed by different parts of the brain, which allows parallel processes^36^; (2) the network functionality associated to the brain activity is interpreted as the collective evaluation of the qubits, layer by layer. Similar to the avalanche behavior in the neural network^35^, the qubits representing the features stimulate a vast number of qubits, denoting the affect and cognition of the agency. Such efficient and rapid affective processing can be manifested in quantum computation^37^.

### Indeterminism in decision-making

The ultimate goal for the Coppélia systems is to make an affect-laden decision based on the observed features and goal states it wants to achieve or avoid. In Silicon Coppélia, where fuzzy sets are employed, decision-making is done by defuzzification through the satisfaction comparison, i.e., selecting the feature with the maximum expected satisfaction [Eq. (S19)] and selecting the action with the maximum expected satisfaction upon that action $i^{(k_{\max })}$ [Eq. (S22)]. Throughout all equations involved in the Silicon Coppélia system (discussed in Section S1), there is no random process. Therefore, given the same features and parameters involved in the evaluation, Silicon Coppélia returns identical decisions. In other words, the processes in Silicon Coppélia are “deterministic.” However, it has been suggested that human behavior has a certain degree of indeterminism^38–40^, i.e., a mix of determinism and indeterminism. Apart from the evidence of quantum processes in physical and information systems of human beings as discussed in Section “Introduction”, more literature suggests the probabilistic nature of human behavior^15,33,41,42^. Such “quantum-like” probabilistic features in decision-making may be found in Q-Coppélia. The probabilistic outcome comes from the measurement of a quantum state in the quantum circuit (in Fig. 11b). As discussed in Section “Vagueness and ambiguity”, in modeling the appraisal kets as two-state systems, the comparison of two qubits involves only 0 and 1. Then, the defuzzification in Silicon Coppélia is translated into a maximum search algorithm as described in Section “Circuit components for quantum logic”, evaluating the probability of each combination, and finally returning an outcome based on the probability distribution via measurement. Therefore, even though the same criteria are applied to Q-Coppélia, the nature of the outcome is entirely different, which is the merit of a quantum algorithm. If similar probabilistic outcomes are to be achieved in conventional algorithms, pseudo-randomness is introduced, where the degree of true randomness as in the physical process may come to one’s attention^43,44^. Quantum logic, however, can naturally simulate such what we believe is realistic behavior.

We represented qubits by Bloch spheres, showing superposition of multiple possibilities represented by algorithms like the solutions in the Schrödinger’s equation. As an approach to decision-making, we had superposed options reduce, or “probabilistically collapse” to definite states as the solution, reflecting randomness observed in decoherence, which is implied in measurement and in proposals for observer effects, causing reduction or collapse. Although in simulations, the Schrödinger’s equation may describe the superposition phase prior to collapse, the mathematics itself certainly does not imply affect, consciousness, or other qualia. Mathematics is a descriptive language. The empirical world we tried to use it for in this paper is still under scrutiny: We suspect that superposition (and collapse) of electrons in the brain—if occurring at all—behaves according to the Schrödinger’s equation (after all, electrons are electrons), carrying information to multiple parts of the brain concurrently. The superposed information distribution and decision-collapse are not a feature of Schrödinger’s mathematics but a conclusion drawn from our assumption about the physics of the brain, which awaits empirical validation still.

We used quantum probability to estimate collapse but, for instance, the mechanism called “objective reduction” or OR mechanism assumes so-called “non-computability,” positing that the choices in OR are selected neither randomly, probabilistically, nor algorithmically^7^. Yet, arguing from Gödel’s theorem, OR as related to quantum gravity would be essential to consciousness^7^. Whereas quantum probability can be simulated, the OR mechanism cannot. Although in the current paper, we did not model “consciousness,” we do have concerns about the OR mechanism escaping verification and validation. If the assumption cannot be simulated, the theory cannot be logically verified while—thus far—not carrying any empirical validation either. Purely pragmatically, then, we wished to model robot behavior that closes in on human conduct and that may be useful even if it turns out that humans do not operate in the way our model assumed.

Vexed question, of course, is where in the brain we may find those assumed quantum effects. We suggested that ion channels in axons may be a candidate but honestly, superposed electrons in microtubules perhaps may be more likely^7^. Current science does not know exactly where in the brain quantum processes take place—if at all.

In and by itself, quantum computing is somewhat futuristic. Physically, it may be possible to manipulate two or three actual qubits whereas our model assumes multiples thereof. As far as we are concerned, something like a conscious robot is a dream still, maybe not even one we want to pursue. Yet, what if the OR mechanism is functional as described in the work of Hameroff et al.^7^, would that enable us to engineer the same mechanism? Could microtubules be seen as small quantum computers, collapsing information by OR to produce “consciousness,” and make conscious decisions? Such quantum computers or “artificial microtubules” may take the form of fullerene nanotubes, graphene, or similar substances, which conceivably may support quantum computing with collapse by OR.

### A comparison of AND and OR between fuzzy and quantum logics

The Zadeh dyadic operators are a common convention in fuzzy logics. The min and max functions are used to represent the and and or operations. In contrast, the use of Toffoli gates in quantum logic implies a different convention of evaluation. To show this, we first assume that the equivalence of some quantity A in fuzzy and quantum logics is characterized by the equality of the value of the membership function and the probability of $|{1} \rangle$, i.e., $\sin ^2\frac{\theta _{\mathrm {A}}}{2}$ with a pure state $\texttt {A}$. Suppose that A and B is evaluated. Whereas the output of A and B in fuzzy logics is given by $\min \bigl( \sin ^2\frac{\theta _{\mathrm {A}}}{2},\sin ^2\frac{\theta _{\mathrm {B}}}{2}\bigr)$, it is $\sin ^2\frac{\theta _{\mathrm {A}}}{2} \sin ^2\frac{\theta _{\mathrm {B}}}{2}$ for quantum logics. Figure S3a and b show the mapping of the and operators using the two types of logics. The fuzzy and shows a rectangular distribution; a ring-like distribution is visible in quantum logic. The rectangular pattern in the fuzzy and is attributed to its definition using the minimum function, namely, $\min \!\left( \texttt {A},\texttt {B}\right) = p$ implies $\texttt {A} = p$ or $\texttt {B} = p$. Similarly, the ring-like distribution originates from the multiplication operation, which gives a reciprocal relation between A and B for the contours, i.e., $\texttt {AB} = p$ implies $\texttt {A} = p/\texttt {B}$. The same shapes of distributions can be found in the or operations for similar reasons (Fig. S3c,d). The result of this exercise is that the value of the membership function of A and B in terms of fuzzy logics is greater than the expectation of the same logical expression in quantum logics. On the contrary, the value of the membership function of A or B in terms of fuzzy logics is less than the expectation of the same logical expression in quantum logics, because $1-\cos ^2\frac{\theta _{\mathrm {A}}}{2}\cos ^2\frac{\theta _{\mathrm {B}}}{2} \ge 1-\cos ^2\frac{\theta _i}{2} = \sin ^2\frac{\theta _i}{2}$ ($i=\mathrm {A},\mathrm {B}$). Put differently, the conjunction rule in quantum logics is stricter than that in fuzzy logics, whereas the disjunction rule in quantum logics is looser than that in fuzzy logics under such convention. As an example, the expectation under evaluation of Eq. (S8) in quantum logic would be generally lower than that in fuzzy logic. The opposite applies to Eq. (S9).

Note that the apparent difference between fuzzy and quantum logics originates primarily from the choice of the convention for the logical operator. In principle, one can also use the min and max comparator as shown in Fig. 3j, instead of the conventional and and or gates in quantum logic. Engineering the rule strength, then, can be done by choosing suitable logical operators. Upon the same perception of the features, goals, and so on, as revealed in observations such as questionnaires, individuals could exhibit various degrees of appraisal of, for instance, relevance and valence. Such variations could be accomplished by tuning the rule strength through the engineering of the logical operators.

The use of logical operators as the building blocks of the model can be further rationalized when interpreting the decision-making process of the Coppélia system as a game aiming at a unanimous choice by conceptualizing the system’s goals as “players”, the features or the choice of actions as strategies, and expected satisfaction values as the payoff. Recently, quantum strategies have been proposed as a stochastic solution of a game that allows enhanced capabilities of judgment for better utility^45–47^. Modeling usually begins with a “bottom-up” formulation of unitary operations that abide by mathematical rules as the strategies of choice^48^. Given a set of preset payoffs for each state of choice and an initial state, the unitary operations are parametrized and optimized for a maximum expected payoff. Such methodology has been employed in classic games such as Prisoner’s dilemma^45,49^, snowdrift^45^, stag-hunt^45,50^, and Kolkata Paise Restaurant^46–48,51^. To compare, the Coppélia system reveals complex affective and cognitive processes during decision-making. It also involves the selection of the dominant feature as an intermediate before choosing the most satisfying action (strategy). These suggest a distinct picture from the typical game study using the bottom-up formulation, leading to a complicated and elaborated construction of the matrices for the strategies. This work resolves the hurdle using a “top-down” approach, starting from the empirically corroborated, psychological-functional side of affective processing, where the building blocks are represented by a set of quantum logics. The quantum gates involved are mostly unitary operations, representing cognitive judgment and perspective interpretation of the information. Mathematically, the construction is similar in nature to the classical formulation—the unitary operations can combine to form huge “master matrices” as strategies. In other words, the algorithm in the manuscript offers an alternative way of modeling quantum strategies by breaking down the factors of affective consideration into logical statements that can be easily translated. The Coppélia system focuses on how a decision arises from satisfaction values, which involve physical processes of psychological appraisals of valence, relevance, use intentions, and an involvement–distance trade-off, i.e., how the payoff values are established. Centering on the cognitive aspects of the agency, such a top-down approach may simulate better humanoid responses in a robot. Such insight opens up an effective route to novel affective robot design by manipulating particular logical blocks instead of directly redesigning the matrices.

## Conclusions and outlook

In search of an appropriate model for contextual affective processing, we worked from the classically modeled Silicon Coppélia system and extended it into a Quantum Coppélia (Q-Coppélia) system^1^. Q-Coppélia adopts quantum algorithms rather than fuzzy logics to improve the simulation of brain processes, including the possibly physical quantum-brain processes. We tried to model counterfactual reasoning for handling the dynamics of contextual impact on behaviors, the causal efficacy of human’s conscious efforts, and so on. We demonstrated how Q-Coppélia can be established from building blocks of quantum circuits and how the algorithms of fuzzy logic can be translated into their quantum counterpart. We found several conceptual advantages of Q-Coppélia. The two-state paradigm together with superposition and mixed states distinguishes an affective state that undergoes an observation from one that does not. Whereas a mix of perspectives may be present in mind, the agency often manifests only one of them during actual decision-making, which we regard as the result of a quantum measurement. This offers a probabilistic rather than a classical deterministic approach for simulating human behavior. The representation of ambiguity is extended not only in the sense that bidimensional unipolar scales are adapted where one may experience positive and negative affect concurrently, as Silicon Coppélia would have it, but also one may have multiple perspectives on one aspect of a dimension of affect, accomplished by the use of mixed states. Rotations of a quantum state in the Bloch sphere are regarded as the transformation of affective states. Quantum entanglement that uses controlled rotations, for instance, effectively allows for mutual interactions among the factors that mediate an output. This facilitates parallel distributed processing in the brain, explaining why affective processing is so fast. Quantum circuits manifest a mix of serial and parallel processes where parallel processing exists in interacting (entangled) and non-interacting networks, and serial processing exists in layered networks of sequential processes. Finally, we remark that there are various Boolean conventions that describe the strength of conditionality in the logical functions. The choice of the convention is intrinsic to the system, not depending on the incoming stimuli. This not only leaves greater freedom in designing robotic systems with various affective and cognitive patterns but also sheds light on representations for various degrees of correlation among different factors that are involved in affective processing. Our work not only demonstrates how a potential candidate of classical affective processing systems may be translated into quantum computing but also reveals a contextual understanding of the relations between quantum algorithms and the fundamental nature of human psychology. The top-down, cognitive aspects-centered modeling approach based on quantum logics provides a conventional paradigm of complex cognitive process formulation, better humanoid responses simulation in a robot, and rational design of affective decision-making in a robot.

A humanoid robot is expected to possess learning capability from its surroundings. In the current manuscript, we did not focus much on the epistemic side of the Q-Coppélia system, but Q-Coppélia does have a module that determines how it perceives the world, what it holds for true or not, and what ontology offers “actionable information” (Fig. 1). The theory that the module relies on is *Epistemics of the Virtual*^﻿52,53^, and the related code is called EpiVir. In the Coppélia system, the machine-learning process is embedded in an encoding process that assesses features in terms of, for instance, ethical qualities as well as epistemics (or “measure of realism”) (Fig. 1). The Epistemics module contains a belief system as the robot’s understanding of the world, the information it uses to undertake action. Sensory inputs go through the ontological classification process composed of a series of logics for determining the ontology, with reference to the belief system. If unexpected or uncertain inputs seem “unrealistic,” a precise and detailed epistemic appraisal is executed, and results are checked for acceptability at various levels of tolerance, allowing for a “learning process” to accept new or modify existing concepts^53^. A learning process like this should be dealing with ambiguity, vagueness, and uncertainty, and quantum logic may be a promising candidate to do so. Centering on the cognitive aspects of the agency, such a top-down approach may simulate better humanoid responses in a robot, which we consider as our future work. Let us embark on further explorations of contextual quantum-decision systems.

## Supplementary Information

Supplementary Information.

## Data Availability

The datasets used and/or analysed during the current study available from the corresponding author on reasonable request.

## References

[1] Hoorn JF, Baier T, Van Maanen JAN, Wester J (2021). Silicon Coppélia and the formalization of the affective process. IEEE Trans. Affect. Comput..

[2] Hoorn, J. F. & Ho, J. K. W. *Robot Affect: The Amygdala as Bloch Sphere*. http://arxiv.org/abs/1911.12128 [cs.AI] (2019).

[3] Bloch F (1946). Nuclear induction. Phys. Rev..

[4] Raghuvanshi A, Perkowski M (2010). Fuzzy quantum circuits to model emotional behaviors of humanoid robots. IEEE Congress Evol. Comput..

[5] Yan F, Iliyasu AM, Liu Z-T, Salama AS, Dong F, Hirota K (2015). Bloch sphere-based representation for quantum emotion space. J. Adv. Comput. Intell. Intell. Inf..

[6] Hameroff SR (2013). Quantum mathematical cognition requires quantum brain biology: The Orch OR theory. Behav. Brain Res..

[7] Hameroff S, Penrose R (2014). Consciousness in the universe: A review of the Orch OR theory. Phys. Life Rev..

[8] Schwartz JM, Stapp HP, Beauregard M (2005). Quantum physics in neuroscience and psychology: A neurophysical model of mind-brain interaction. Philos. Trans. R. Soc. B.

[9] Narens, L. On replacing “quantum thinking” with counterfactual reasoning. in *Contextuality from Quantum Physics to Psychology*, 323 (2016). 10.1142/9789814730617_0013

[10] Tversky A, Kahneman D (1974). Judgment under uncertainty: Heuristics and biases. Science.

[11] Khrennikov A, Asano M (2020). A quantum-like model of information processing in the brain. Appl. Sci..

[12] Tversky A, Kahneman D (1983). Extensional versus intuitive reasoning: The conjunction fallacy in probability judgment. Psychol. Rev..

[13] Pothos EM, Busemeyer JR (2009). A quantum probability explanation for violations of rational decision theory. Proc. R. Soc. B.

[14] Busemeyer JR, Pothos EM, Franco R, Trueblood JS (2011). A quantum theoretical explanation for probability judgment errors. Psychol. Rev..

[15] Wang Z, Solloway T, Shiffrin RM, Busemeyer JR (2014). Context effects produced by question orders reveal quantum nature of human judgments. Proc. Natl. Acad. Sci. USA.

[16] Khrennikova P, Haven E, Khrennikov A (2014). An application of the theory of open quantum systems to model the dynamics of party governance in the US political system. Int. J. Theor. Phys..

[17] Asano M, Basieva I, Khrennikov A, Ohya M, Tanaka Y (2017). A quantum-like model of selection behavior. J. Math. Psychol..

[18] Asano M, Basieva I, Khrennikov A, Yamato I (2017). A model of differentiation in quantum bioinformatics. Prog. Biophys. Mol. Biol..

[19] Yearsley JM, Busemeyer JR (2016). Quantum cognition and decision theories: A tutorial. J. Math. Psychol..

[20] Russell JA, Carroll JM (1999). On the bipolarity of positive and negative affect. Psychol. Bull..

[21] Russell JA, Carroll JM (1999). The phoenix of bipolarity: Reply to Watson and Tellegen (1999). Psychol. Bull..

[22] Scherer KR (2005). What are emotions? And how can they be measured?. Soc. Sci. Inf..

[23] Yan F, Iliyasu AM, Jiao S, Yang H (2019). Quantum structure for modelling emotion space of robots. Appl. Sci..

[24] Yan F, Iliyasu AM, Hirota K (2021). Conceptual framework for quantum affective computing and its use in fusion of multi-robot emotions. Electronics.

[25] Yan F, Yang X, Li N, Yu X, Zhai H (2021). Emotion generation and transition of companion robots based on Plutchik’s model and quantum circuit schemes. Secur. Commun. Netw..

[26] Ling, X., Zhao, S. & Zhai, H. Quantum representation for robot’s emotions based on PAD model. in *The 7th International Workshop on Advanced Computational Intelligence and Intelligent Informatics* (IWACIII 2021, M1-7–1, 2021).

[27] LeDoux JE (1998). The Emotional Brain: The Mysterious Underpinnings of Emotional Life.

[28] Crone EA, Konijn EA (2018). Media use and brain development during adolescence. Nat. Commun..

[29] Cerić F (2012). Fast route versus slow route: Electrophysiological and behavioural evidences of emotional processing pathways. Estud. Psicol..

[30] Oatley K (2019). The human unconscious in evolution. Psychol. Inq..

[31] Hoorn, J. F., Baier, T., van Maanen, J. A. N., Wester, J. & Offermans, M. *Silicon Coppélia: An implementation in Ptolemy*. https://bitbucket.org/robopop/silicon-coppelia (2016). Accessed 26 Jan 2022.

[32] Hoorn JF, Konijn EA, Pontier MA (2019). Dating a synthetic character is like dating a man. Int. J. Soc. Robot..

[33] Busemeyer JR, Wang Z (2015). What is quantum cognition, and how is it applied to psychology?. Curr. Dir. Psychol. Sci..

[34] Dawson MRW (2013). Mind, Body, World: Foundations of Cognitive Science.

[35] Flusberg, S. J. & McClelland, J. L. Connectionism and the emergence of mind. in *Contextuality from Quantum Physics to Psychology* (ed Chipman, S. E. F.) (2017). 10.1093/oxfordhb/9780199842193.013.5

[36] Fusar-Poli P (2009). Functional atlas of emotional faces processing: A voxel-based meta-analysis of 105 functional magnetic resonance imaging studies. J. Psychiatry Neurosci..

[37] Dalla Chiara ML, Giuntini R, Leporini R, Negri E, Sergioli G (2015). Quantum information, cognition, and music. Front. Psychol..

[38] Cziko GA (1989). Unpredictability and indeterminism in human behavior: Arguments and implications for educational research. Educ. Res..

[39] Neuringer A (1986). Can people behave randomly? The role of feedback. J. Exp. Psychol. Gen..

[40] Song C, Qu Z, Blumm N, Barabási A-L (2010). Limits of predictability in human mobility. Science.

[41] Li J-A (2020). Quantum reinforcement learning during human decision-making. Nat. Hum. Behav..

[42] Bruza PD, Wang Z, Busemeyer JR (2015). Quantum cognition: A new theoretical approach to psychology. Trends Cogn. Sci..

[43] Jacak MM, Jóźwiak P, Niemczuk J, Jacak JE (2021). Quantum generators of random numbers. Sci. Rep..

[44] Bird JJ, Ekárt A, Faria DR (2020). On the effects of pseudorandom and quantum-random number generators in soft computing. Soft Comput..

[45] Pawela L (2016). Quantum games on evolving random networks. Phys. A: Stat. Mech. Appl..

[46] Park, T. & Saad, W. Kolkata paise restaurant game for resource allocation in the internet of things. in *2017 51st Asilomar Conference on Signals, Systems, and Computers*, 1774–1778. 10.1109/ACSSC.2017.8335666 (2017).

[47] Sharif P, Heydari H (2012). Strategies in a symmetric quantum Kolkata restaurant problem. AIP Conf. Proc..

[48] Chakrabarti BK, Rajak A, Sinha A (2022). Stochastic learning in Kolkata paise restaurant problem: Classical and quantum strategies. Front. Artif. Intell. Appl..

[49] Li A, Yong X (2014). Entanglement guarantees emergence of cooperation in quantum prisoner’s dilemma games on networks. Sci. Rep..

[50] Hanauske M, Bernius S, Dugall B (2007). Quantum game theory and open access publishing. Phys. A: Stat. Mech. Appl..

[51] Ramzan M (2013). Three-player quantum Kolkata restaurant problem under decoherence. Quant. Inf. Process..

[52] Hoorn JF (2012). Epistemics of the Virtual.

[53] Hoorn JF, Tuinhof DJ (2022). A robot’s sense-making of fallacies and rhetorical tropes: Creating ontologies of what humans try to say. Cogn. Syst. Res..

[54] Hoorn, J. F. Psychological aspects of technology interacting with humans. in *The Handbook of the Psychology of Communication Technology*, 176–201 (Wiley, 2015). 10.1002/9781118426456.ch8.

