# Supplementary material for: Quantum affective processes for multidimensional decision-making
Source: Sci Rep. 2022 Nov 28;12:20468. doi: 10.1038/s41598-022-22855-0 (PMC9705568; doi:10.1038/s41598-022-22855-0)
Supplement: Supplementary file 1 — Supplementary Information. [file 41598_2022_22855_MOESM1_ESM.pdf]

Supplementary Information

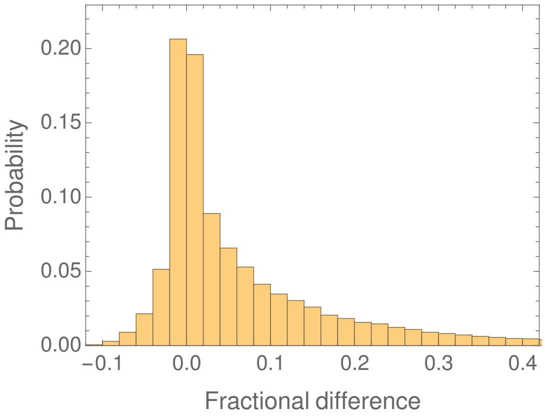

(a) Dual-conditioned R_y rotation

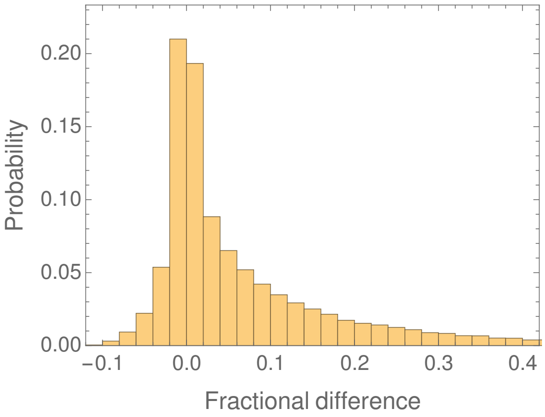

(b) Triple-conditioned R_y rotation

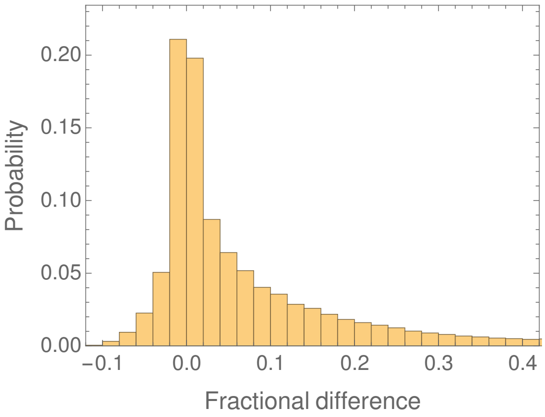

(c) Quadruple-conditioned R_y rotation

Figure S1: Simulated fractional (relative) difference between the weighted mean (or inner product) determined by the classical fuzzy-based Silicon Coppélia system and quantum logics. The scenario in Eq. (S11) is taken in this simulation. The quantum circuit consists of successive single-control C-R_y rotations, as depicted in Eq. (3) and Fig. 3h. Each condition is made up of one distinct weight factor $|\phi_i\rangle = \cos\frac{\phi_i}{2}|0\rangle + \sin\frac{\phi_i}{2}|1\rangle$, where $\phi_i \in [0, \pi]$ and $\sum_i \phi_i = \pi$. The angle of rotation $\alpha_i\pi$ for each condition is arbitrary with $\alpha_i \in [0, \pi]$ and $\sum_i \alpha_i = 1$. 50,000 combinations of ϕ_i and α_i were randomly generated to evaluate the resultant target state $|\psi\rangle$. The probability of observing state $|1\rangle$ is compared to its classical counterpart, which is given by $\sum_i \sin^2\frac{\alpha_i\pi}{2}\sin^2\frac{\phi_i}{2}$. A positive relative difference implies a greater value obtained by quantum logic. A single-conditioned C-R_y rotation gives exact results. The distributions for C-R_y rotations under 2–4 control qubits are similar, with a high proportion showing little deviation.

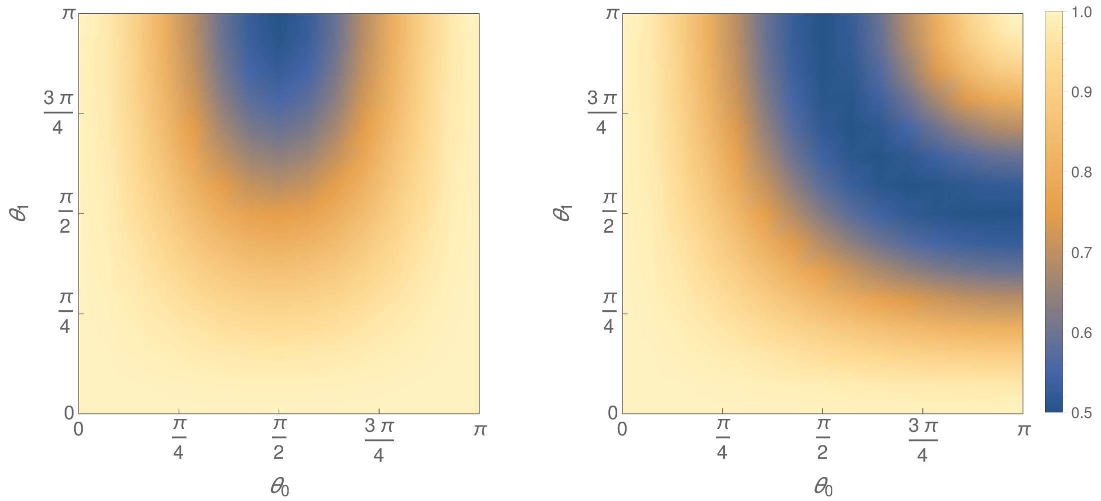

(a) Rotation formalism (Fig. 3f) with $\theta_1 = \alpha\pi$

(b) Entanglement formalism (Fig. 3g) with $|\alpha\rangle = \cos\frac{\theta_1}{2}|0\rangle + \sin\frac{\theta_1}{2}|1\rangle$

Figure S2: The purity of the target state $\gamma = \mathrm{Tr}\left(\hat{\rho}^2\right)$ under the two-state generation formalisms with an additional control qubit $\cos\frac{\theta_0}{2}|0\rangle + \sin\frac{\theta_0}{2}|1\rangle$. $\gamma = 0.5$ represents a fully mixed state.

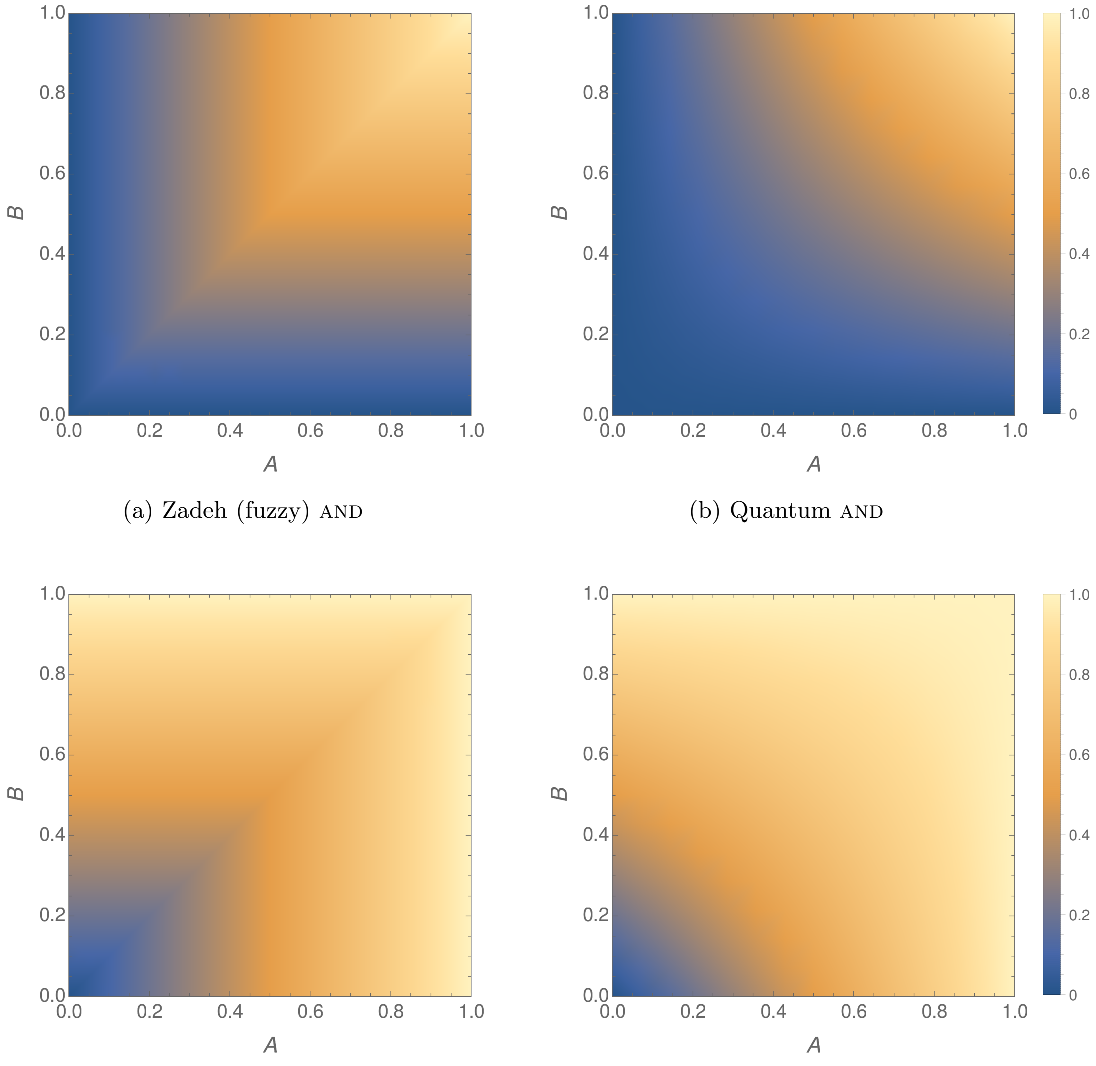

(a) Zadeh (fuzzy) AND

(b) Quantum AND

(c) Zadeh (fuzzy) OR

(d) Quantum OR

Figure S3: The output of the AND (a,b) and OR (c,d) gates using the conventional Zadeh operators for fuzzy logics (a,c) or the quantum logic operators (b,d). For the quantum operators, the values represent the probability of $|1\rangle$.

S1 Silicon Coppélia and Terminology

S1.1 Encoding

During *encoding* the features of a human or virtual agency, each feature is numbered k in a set $\mathscr{F}$, for their level of *ethics* (good $\mathcal{E}^+$ or bad $\mathcal{E}^-$), *aesthetics* (beautiful $\mathcal{S}^+$ or ugly $\mathcal{S}^-$), and *epistemics* (realistic $\mathcal{P}^+$ or unrealistic $\mathcal{P}^-$). During the encoding, moreover, the robot evaluates how far the user has certain *affordances* (aids $\mathcal{F}^+$ or obstacles $\mathcal{F}^-$), action possibilities that make the other agency functional as a tool to achieve robot goals or not (e.g., to recharge its battery, to keep it from falling, to serve its user). These dimensions constitute a set of appraisal variables $\mathscr{D} = \{\mu = \mathcal{E}, \mathcal{F}, \mathcal{S}, \mathcal{P}\} \times \{\nu = +, -\} = \{\mu^\nu\}$. Features receive different feature weights $w^{(k)}$ to represent subjective judgments of any kind (weights are unconstrained). Each appraisal variable also has its appraisal weight for the feature $d_l^{(k)}$ ($l = \mu^\nu \in \mathscr{D}$) as an indicator of its dominance in the appraisal process. Empirical research found that features pertaining to ethics and affordances are better predictors of use intentions and engagement than epistemic aspects (e.g., the agency is real or virtual) or aesthetics, i.e., $d_{\mathcal{E}\pm}^{(k)}, d_{\mathcal{F}\pm}^{(k)} > d_{\mathcal{P}\pm}^{(k)}, d_{\mathcal{S}\pm}^{(k)}$.[1] The encoding process generates the perceived weight of the appraisal variables

$$p_l^{(k)} = w^{(k)} d_l^{(k)}, \qquad l = \mu^\nu \in \mathscr{D}. \tag{S1}$$

Table S1 summarizes the parameters used in the encoding process.

Table S1: Variables involved in feature encoding of the Silicon Coppélia system, and their corresponding kets in the Quantum Coppélia system.

Name	Si-Coppélia notation	Description	Qubit notation
Set of features	$\mathscr{F}$	$\mathscr{F} = \{k = 1, \cdots, N_\mathrm{k}\} \subseteq \Omega$	
Set of all possible features	Ω	$\mathscr{F} \subseteq \Omega$	
Feature	k	$k \in \mathscr{F}$	

Table S1: Variables involved in feature encoding of the Silicon Coppélia system, and their corresponding kets in the Quantum Coppélia system. (Cont'd)

Name	Si-Coppélia notation	Description	Qubit notation
Set of appraisal variable labels	$\mathscr{D}$	$\mathscr{D} = \{\mu = \mathcal{E}(eth), \mathcal{F}(aff), \mathcal{S}(aest), \mathcal{P}(ep)\} \times \{\nu = +, -\} = \{\mu^\nu\}$<br>$\mathcal{E}^+ = good$, $\mathcal{E}^- = bad$,<br>$\mathcal{F}^+ = aid$, $\mathcal{F}^- = obstacle$,<br>$\mathcal{S}^+ = beautiful$, $\mathcal{S}^- = ugly$,<br>$\mathcal{P}^+ = real$, $\mathcal{P}^- = unreal$	
Appraisal weight	$\vec{d}$	$\vec{d} = \left(\vec{d}^{(k)}\right)_{k \in \mathscr{F}}$	$\left\|d^{(k)}_{\mathcal{E}+}\right\rangle$, $\left\|d^{(k)}_{\mathcal{E}-}\right\rangle$,<br>$\left\|d^{(k)}_{\mathcal{F}+}\right\rangle$, $\left\|d^{(k)}_{\mathcal{F}-}\right\rangle$,<br>$\left\|d^{(k)}_{\mathcal{S}+}\right\rangle$, $\left\|d^{(k)}_{\mathcal{S}-}\right\rangle$,<br>$\left\|d^{(k)}_{\mathcal{P}+}\right\rangle$, $\left\|d^{(k)}_{\mathcal{P}-}\right\rangle$
Appraisal weight for feature k	$\vec{d}^{(k)}$	$\vec{d}^{(k)} = \left(d^{(k)}_l\right)_{l=\mu^\nu \in \mathscr{D}}$<br>$d^{(k)}_l \in [0, 1]$	
Feature weight	$\vec{w}$	$\vec{w} = \left(w^{(k)}\right)_{k \in \mathscr{F}}, w^{(k)} \in [0, 1]$	$\left\|w^{(k)}\right\rangle$
Perceived weight of appraised variable	$\vec{p}$	$\vec{p} = \left(\vec{p}^{(k)}\right)_{k \in \mathscr{F}}$<br>$\vec{p}^{(k)} = w^{(k)}\vec{d}^{(k)} = \left(p^{(k)}_l\right)_{l \in \mathscr{D}} = \left(w^{(k)}\vec{d}^{(k)}\right)_{l \in \mathscr{D}}$<br>$p^{(k)}_l \in [0, 1]$	$\left\|p^{(k)}_{\mathcal{E}+}\right\rangle$, $\left\|p^{(k)}_{\mathcal{E}-}\right\rangle$,<br>$\left\|p^{(k)}_{\mathcal{F}+}\right\rangle$, $\left\|p^{(k)}_{\mathcal{F}-}\right\rangle$,<br>$\left\|p^{(k)}_{\mathcal{S}+}\right\rangle$, $\left\|p^{(k)}_{\mathcal{S}-}\right\rangle$,<br>$\left\|p^{(k)}_{\mathcal{P}+}\right\rangle$, $\left\|p^{(k)}_{\mathcal{P}-}\right\rangle$

S1.2 Comparison

In the *comparison* phase, the features are judged for *relevance* to robot goals j in a set of goals $\mathscr{G}$ (relevant ($R^{(k)}$) or irrelevant ($R^{\dagger(k)}$)) and *valence* to robot goals (positive or negative outcome expectancies ($V^{(k)}$ or $V^{\dagger(k)}$)) using the membership functions. Relevance determines the intensity of the affective response, whereas valence determines its direction. The variables involved in the comparison phase are summarized in Table S2. An agency's features (e.g., its intentions) encoded as positive (e.g., "good") may afford the facilitation of a desired robot goal, for example, to have maintenance every once in a while. This instigates positive outcome expectancies. When the features are found to be highly relevant to the robot goals (for either positive or negative outcome expectancies), a high level of relevance results.

Table S2: Variables involved in the relevance and valence evaluation as well as their related action tendencies in the Silicon Coppélia system, and their corresponding kets in the Quantum Coppélia system.

Name	Si-Coppélia notation	Description	Qubit notation
Set of choices of action	$\mathscr{A}^{(k)}$	$\mathscr{A}^{(k)} = \left\{i^{(k)}\right\}$	
Choice of action for feature k	$i^{(k)}$	$t\left(i^{(k)}\right) \in \mathscr{T}$	
Action tendency	$\mathscr{T}$	$\mathscr{T} = \left\{\textit{positive}, \textit{negative}, \textit{change}, \textit{avoid}\right\}$	
Set of goals	$\mathscr{G}$	$\mathscr{G} = \{j = 1, ..., N_\mathrm{j}\}$	
Goal	j	$j \in \mathscr{G}$	
Ambition towards goal	$\vec{a}$	$\vec{a} = (a_j \in [-1,1])_{j\in\mathscr{G}}$<br>$a_j > 0 \Rightarrow \textit{desired}$<br>$a_j < 0 \Rightarrow \textit{undesired}$	$\left\|a_j^+\right\rangle$: *desired*<br>$\left\|a_j^-\right\rangle$: *undesired*
Belief for feature k	$\vec{b}^{(k)}$	$\vec{b}^{(k)} = \left(\vec{b}_j^{(k)}\right)_{j\in\mathscr{G}}$<br>$\vec{b}_j^{(k)} = \left(b_{ij}^{(k)} \in [-1,1]\right)_{i\in\mathscr{A}^{(k)}}$<br>$b_{ij}^{(k)} > 0 \Rightarrow \textit{facilitate}$<br>$b_{ij}^{(k)} < 0 \Rightarrow \textit{inhibit}$	$\left\|b_{i+j}^{(k)}\right\rangle$: *facilitate*<br>$\left\|b_{i-j}^{(k)}\right\rangle$: *inhibit*
Membership function of x belonging to $\mathscr{Y}$	$\mu_{\mathscr{Y}}(x)$	$\mu_{\mathscr{Y}}(x) \in [0,1]$	
Affect (goal j by action i)	$\mu_\mathrm{A}\left(b_{ij}^{(k)}\right)$	*affects* = *facilitates* OR *inhibits*	$\left\|A_{ij}^{(k)}\right\rangle$
Important (for goal j)	$\mu_\mathrm{I}(a_j)$	*important* = *desired* OR *undesired*	$\left\|I_j\right\rangle$
Agreement to any action for goal j for feature k (for relevance)	$g_j^{(k)}$	$g_j^{(k)} \in [-1,1]$<br>$g_j^{(k)} > 0 \Rightarrow \textit{agree}$<br>$g_j^{(k)} < 0 \Rightarrow \textit{disagree}$	$\left\|g_j^{(k)}\right\rangle$: *agree*<br>$\left\|g_j^{\dagger(k)}\right\rangle$: *disagree*
Relevance of encoded features	$R_\mathrm{p}^{(k)}$	$R_\mathrm{p}^{(k)} = \mu_\mathrm{R}\left(\vec{p}^{(k)}\right)$<br>$R_\mathrm{p}^{(k)} \in [0,1]$	$\left\|R_\mathrm{p}^{(k)}\right\rangle$

Table S2: Variables involved in the relevance and valence evaluation as well as their related action tendencies in the Silicon Coppélia system, and their corresponding kets in the Quantum Coppélia system. (Cont'd)

Name	Si-Coppélia notation	Description	Qubit notation
Relevance & irrelevance from goal comparison	Relevance $\vec{R}^{(k)} = \big(R_j^{(k)}\big)$, Irrelevance $\vec{R}^{\dagger(k)} = \big(R_j^{\dagger(k)}\big)$	$R_j^{(k)} = \mu_{\mathrm{R}}\big(\vec{b}^{(k)}, a_j, g_j^{(k)}\big)$, $R_j^{\dagger(k)} = \mu_{\mathrm{R}^\dagger}\big(\vec{b}^{(k)}, a_j, g_j^{(k)}\big)$, $R_j^{(k)}, R_j^{\dagger(k)} \in [0,1]$	$\left\vert R_j^{(k)}\right\rangle$: *relevance* $\left\vert R_j^{\dagger(k)}\right\rangle$: *irrelevance*
Relevance & irrelevance	$R^{(k)}$: Relevance $R^{\dagger(k)}$: Irrelevance	$R^{(k)} = \mu_{\mathrm{R}}\big(\vec{R}^{(k)}, R_{\mathrm{P}}^{(k)}\big)$, $R^{\dagger(k)} = \mu_{\mathrm{R}^\dagger}\big(\vec{R}^{(k)}, R_{\mathrm{P}}^{(k)}\big)$, $R^{(k)}, R^{\dagger(k)} \in [0,1]$	$\left\vert R^{(k)}\right\rangle$: *relevance* $\left\vert R^{\dagger(k)}\right\rangle$: *irrelevance*
Agreement to action i for goal j for feature k (for valence)	$g'^{(k)}_{ij}$	$\vec{g}'^{(k)} = \big(g'^{(k)}_{ij}\big)_{i\in\mathscr{A}^{(k)}, j\in\mathscr{G}}$ $g'^{(k)}_{ij} \in [-1,1]$ $g'^{(k)}_{ij} > 0 \Rightarrow$ *agree* $g'^{(k)}_{ij} < 0 \Rightarrow$ *disagree*	$\left\vert g'^{(k)}_{ij}\right\rangle$: *agree* $\left\vert g'^{\dagger(k)}_{ij}\right\rangle$: *disagree*
Valence	$V^{(k)}$ (+), $V^{\dagger(k)}$ (−)	$V^{(k)} = \mu_{\mathrm{V}}\big(p_{\mathcal{E}\pm}^{(k)}, p_{\mathcal{F}\pm}^{(k)}, \vec{b}^{(k)}, \vec{a}, \vec{g}'^{(k)}\big)$, $V^{\dagger(k)} = \mu_{\mathrm{V}^\dagger}\big(p_{\mathcal{E}\pm}^{(k)}, p_{\mathcal{F}\pm}^{(k)}, \vec{b}^{(k)}, \vec{a}, \vec{g}'^{(k)}\big)$, $V^{(k)}, V^{\dagger(k)} \in [0,1]$	$\left\vert V^{(k)}\right\rangle$: *+ve valence* $\left\vert V^{\dagger(k)}\right\rangle$: *−ve valence*

S1.2.1 Relevance

Specifically, relevance and irrelevance are evaluated from a set of (1) goal-and-action–based rules and (2) appraisal-variables–based rules. For the former, an action $i^{(k)}$ for feature k may be chosen from a set of choice of action $\mathscr{A}^{(k)}$ where $i^{(k)}$ can be mapped to some action tendency in a set $\mathscr{T}$ via $t : \mathscr{A}^{(k)} \to \mathscr{T}$ (see *Satisfaction* in Section S1.3.3 for details). On the other hand, a goal j from a set $\mathscr{G}$, *desired* or *undesired*, defines what the agency wants to achieve or avoid. The goal is *important* whether it is desired or undesired. The action $i^{(k)}$ has an effect on (*affects*) goal j if it either *facilitates* or *inhibits* the goal. Two statements concerning a particular feature k can be set up: ζ_1: ANY $i^{(k)} \in \mathscr{A}^{(k)}$ affects goal j, and ζ_2: goal j is important. The agency may *agree* or *disagree* to ζ_1 and ζ_2. *Agree* and *disagree* act as a bidimensional unipolar scale as one can simultaneously agree to the statements in some aspects and

disagree in other aspects. The three statements result in the following rule:

$$\begin{aligned}
&\text{IF} \quad \Big(\zeta_1 : \bigvee_{i^{(k)} \in \mathscr{A}^{(k)}} \big(\text{action } i^{(k)} \text{ affects goal } j\big)\Big) \\
&\qquad \bigwedge \quad \zeta_2 : \text{goal } j \text{ is important} \\
&\qquad \bigwedge \quad \zeta_3 : \text{agree to } \ (\zeta_1 \wedge \zeta_2) \\
&\text{THEN} \quad \text{feature } k \text{ is relevant (under goal } j\text{)},
\end{aligned} \tag{S2}$$

or, abbreviated in terms of membership function, $R_j^{(k)} = \mu_{\mathrm{R}}\big(\vec{b}^{(k)}, a_j, g_j^{(k)}\big)$, where $R_j^{(k)}$ is the value of relevance under goal j and μ_{R} is the corresponding membership function. For the appraisal-variables–based rules, the agency checks separately the four appraisal variables. If the feature possesses either the indicative or the counter-indicative appraisal property, the feature is considered relevant. Considering all appraisal variables, the rule becomes

$$\begin{aligned}
&\text{IF} \quad \zeta_4 : \bigvee_{l \in \mathscr{D}} \text{feature } k \text{ is } l \\
&\text{THEN} \quad \text{feature } k \text{ is relevant (due to encoded features)},
\end{aligned} \tag{S3}$$

or, abbreviated in terms of membership function, $R_{\mathrm{p}}^{(k)} = \mu_{\mathrm{R}}\big(\vec{p}^{(k)}\big)$, where $R_{\mathrm{p}}^{(k)}$ is the value of relevance due to encoded features. The disjunction of the above 2 rules (Eqs. (S2) and (S3)) for all goals gives the resultant relevance, i.e,

$$\begin{aligned}
&\text{IF} \quad \bigvee_{j \in \mathscr{G}} \Big(\text{feature } k \text{ is relevant under goal } j\Big) \\
&\qquad \bigvee \quad \text{feature } k \text{ is relevant due to encoded features} \\
&\text{THEN} \quad \text{feature } k \text{ is relevant},
\end{aligned} \tag{S4}$$

or, abbreviated in terms of membership function, $R^{(k)} = \mu_{\mathrm{R}}\big(\vec{R}^{(k)}, R_{\mathrm{p}}^{(k)}\big)$, where $R^{(k)}$ is the value of relevance and $\vec{R}^{(k)} = \big(R_j^{(k)}\big)$. The two sets of rules are similar for irrelevance evaluation. The negation of the conditions leads to irrelevance. Nonetheless, for *agree* and *disagree* as a pair of bidimensional unipolar scales (one can simultaneously agree and disagree to one entity given multiple perspectives), we consider

the negation of *agree* to be *disagree*. Therefore,

$$
\begin{aligned}
\textsc{if} \quad & \neg\Bigg[\Big(\zeta_1 : \bigvee_{i^{(k)} \in \mathscr{A}^{(k)}} \big(\text{action } i^{(k)} \text{ affects goal } j\big)\Big) \\
& \quad \bigwedge \quad \Big(\zeta_2 : \text{ goal } j \text{ is important}\Big)\Bigg] \\
& \bigvee \quad \zeta_3^\dagger : \text{disagree to } (\zeta_1 \wedge \zeta_2) \\
\textsc{then} \quad & \text{feature } k \text{ is irrelevant (under goal } j),
\end{aligned}
\tag{S5}
$$

or, abbreviated in terms of membership function, $R_j^{\dagger(k)} = \mu_{\mathrm{R}^\dagger}\big(\vec{b}^{(k)}, a_j, g_j^{(k)}\big)$, where $R_j^{\dagger(k)}$ is the value of relevance under goal j and $\mu_{\mathrm{R}^\dagger}$ is the corresponding membership function, and

$$
\begin{aligned}
\textsc{if} \quad & \neg\Big(\zeta_4 : \bigvee_{l \in \mathscr{D}} \text{feature } k \text{ is } l\Big) \\
\textsc{then} \quad & \text{feature } k \text{ is irrelevant (due to feature encoding).}
\end{aligned}
\tag{S6}
$$

Note that the value of irrelevance due to encoded features is $1 - R_{\mathrm{p}}^{(k)}$. The disjunction of the above 2 rules (Eqs. (S5) and (S6)) gives the resultant irrelevance, i.e.,

$$
\begin{aligned}
\textsc{if} \quad & \bigvee_{j \in \mathscr{G}} \Big(\text{feature } k \text{ is irrelevant under goal } j\Big) \\
& \bigvee \text{ feature } k \text{ is irrelevant due to encoded features} \\
\textsc{then} \quad & \text{feature } k \text{ is irrelevant,}
\end{aligned}
\tag{S7}
$$

or, abbreviated in terms of membership function, $R^{\dagger(k)} = \mu_{\mathrm{R}^\dagger}\big(\vec{R}^{\dagger(k)}, R_{\mathrm{p}}^{(k)}\big)$, where $R^{\dagger(k)}$ is the value of irrelevance and $\vec{R}^{\dagger(k)} = \big(R_j^{\dagger(k)}\big)$.

S1.2.2 Valence

Valence evaluation relies primarily on the available beliefs ($b_{ij}^{(k)}$) of whether an action $i^{(k)}$ facilitates ($b_{ij}^{(k)} > 0$) or inhibits ($b_{ij}^{(k)} < 0$) the goal, the ambition to the goal (a_j) (desired ($a_j > 0$) or undesired ($a_j < 0$)), and the agreement ($g'^{(k)}_{ij}$) of the agency to the former two statements (agree ($g'^{(k)}_{ij} > 0$) or disagree ($g'^{(k)}_{ij} < 0$)). Depending on the combination of facilitates/inhibits, desired/undesired and

agree/disagree, the positive and negative valence are evaluated as follows:

$$
\begin{aligned}
&\text{IF} && \zeta_1' : \text{feature } k \text{ is } \begin{Bmatrix} \text{aid} \\ \text{obstacle} \end{Bmatrix} \\
& && \bigwedge \ \zeta_2' : \text{feature } k \text{ is } \begin{Bmatrix} \text{good} \\ \text{bad} \end{Bmatrix} \\
& && \bigwedge \ \zeta_3' : \text{action } i^{(k)} \begin{Bmatrix} \text{facilitates}, & \operatorname{sgn}\big(b_{ij}^{(k)}\big) = +1 \\ \text{inhibits}, & \operatorname{sgn}\big(b_{ij}^{(k)}\big) = -1 \end{Bmatrix} \text{ goal } j \\
& && \bigwedge \ \zeta_4' : \text{goal } j \text{ is } \begin{Bmatrix} \text{desired}, & \operatorname{sgn}\big(a_j\big) = +1 \\ \text{undesired}, & \operatorname{sgn}\big(a_j\big) = -1 \end{Bmatrix} \\
& && \bigwedge \ \zeta_5' : \begin{Bmatrix} \text{agree}, & \operatorname{sgn}\big(g'^{(k)}_{ij}\big) = +1 \\ \text{disagree}, & \operatorname{sgn}\big(g'^{(k)}_{ij}\big) = -1 \end{Bmatrix} \text{ to } (\zeta_1' \wedge \zeta_2' \wedge \zeta_3' \wedge \zeta_4') \\
&\text{THEN} && \text{feature } k \text{ has } \begin{Bmatrix} \text{positive}, & \operatorname{sgn}\big(b_{ij}^{(k)} a_j g'^{(k)}_{ij}\big) = +1 \\ \text{negative}, & \operatorname{sgn}\big(b_{ij}^{(k)} a_j g'^{(k)}_{ij}\big) = -1 \end{Bmatrix} \text{ valence (under goal } j \text{ and action } i^{(k)})
\end{aligned}
\tag{S8}
$$

where $\operatorname{sgn}(x)$ is the sign function. For a feature with 4 possible actions, Eq. (S8) is a condensation of $2 \times 2 \times (4 \times 2) \times 2 \times 2 = 128$ logical statements per goal per feature, containing 64 for each positive and negative valence. The resultant positive or negative valence is the disjunction of the respective statements, i.e.,

$$
\begin{aligned}
&\text{IF} && \bigvee_{j \in \mathscr{G}} \bigvee_{i^{(k)} \in \mathscr{A}^{(k)}} \left(\text{feature } k \text{ has } \begin{Bmatrix} \text{positive} \\ \text{negative} \end{Bmatrix} \text{ valence under goal } j \text{ and action } i^{(k)} \right) \\
&\text{THEN} && \text{feature } k \text{ has } \begin{Bmatrix} \text{positive} \\ \text{negative} \end{Bmatrix} \text{ valence,}
\end{aligned}
\tag{S9}
$$

or, abbreviated in terms of membership function, $V^{(k)} = \mu_{\mathrm{V}}\big(p^{(k)}_{\mathcal{E}\pm}, p^{(k)}_{\mathcal{F}\pm}, \vec{b}^{(k)}, \vec{a}, \vec{g}'^{(k)}\big)$, where $V^{(k)}$ is the value of positive valence, and $V^{\dagger(k)} = \mu_{\mathrm{V}^\dagger}\big(p^{(k)}_{\mathcal{E}\pm}, p^{(k)}_{\mathcal{F}\pm}, \vec{b}^{(k)}, \vec{a}, \vec{g}'^{(k)}\big)$, where $V^{\dagger(k)}$ is the value of negative valence. μ_{V} and $\mu_{\mathrm{V}^\dagger}$ are the corresponding membership functions.

The measures in the encode phase – mediated by relevance and valence in the comparison phase and moderated by *similarity* between the features of robot and user (similar or dissimilar) – determine the robot's responses Fig. 1.[1] However, empirical research showed that relevance and valence are more important to engagement than similarity.[1] For the sake of simplicity, we modeled relevance and valence alone.

S1.3 Response

In the response phase, the robot generates rational cognitions about use intentions and runs affective processes for engagement. Cognition and affect are then collectively considered to estimate the expected satisfaction. Decision-making is done based on the expected satisfaction upon various response strategies (positive and negative approach, change, avoid, etc.)

S1.3.1 Use intentions

In the *response* phase, the robot calculates a value for the so-called *use intentions*, the willingness to employ the user or another agency as a means to achieve robot goals. The variables involved in the evaluation of use intentions are summarized in Table S3. *Utility* as an intermediate variable to indicate the usefulness of the feature to the agency with respect to its goals is calculated first. The expected utility is given by

$$u'^{(k)}_{ij} = b^{(k)}_{ij} a_j. \tag{S10}$$

The *mean expected utility* over goals for an action $i^{(k)}$ can then be calculated, subject to a weight for each goal $\Theta^{(k)}\big(u'^{(k)}_{ij}\big)$:

$$\bar{u}'^{(k)}_i = \frac{\sum_{j \in \mathscr{G}} \Theta^{(k)}\big(u'^{(k)}_{ij}\big) \cdot u'^{(k)}_{ij}}{\sum_{j \in \mathscr{G}} \Theta^{(k)}\big(u'^{(k)}_{ij}\big)}. \tag{S11}$$

Then, the *indicative utility* $(u'^{(k)}_{+})$ and *counter-indicative utility* $(u'^{(k)}_{-})$ values of the feature k are determined by the highest and lowest mean utility for the action $i^{(k)}$:

$$u'^{(k)}_{\pm} = \max_{i^{(k)} \in \mathscr{A}^{(k)}} \big(\pm \bar{u}'^{(k)}_i, 0\big). \tag{S12}$$

Use intentions are modeled as a complex composition of various factors, each carrying a particular weight. The indicative $(u^{(k)}_{+})$ and counter-indicative $(u^{(k)}_{-})$ use intentions can be evaluated by

$$\vec{u}^{(k)} = \begin{bmatrix} u^{(k)}_{+} \\ u^{(k)}_{-} \end{bmatrix} = \mathbf{B}_{\mathrm{ui}}\, \vec{r}^{(k)}_{\mathrm{ui}} \tag{S13}$$

where the 2×10 matrix $\mathbf{B}_{\mathrm{ui}}$ is the weight matrix for use intentions, containing the weights for $\vec{r}^{(k)}_{\mathrm{ui}}$, the component vector for use intention in $\mathbb{R}^{10}$:

$$\vec{r}^{(k)}_{\mathrm{ui}} = \mathrm{vec}\left(\left\{ \begin{bmatrix} u'^{(k)}_{+} \\ u'^{(k)}_{-} \end{bmatrix}, \quad \begin{bmatrix} u'^{(k)}_{+} \\ u'^{(k)}_{-} \end{bmatrix} \otimes \begin{bmatrix} s & p^{(k)}_{\mathcal{S}+} \\ s^{\dagger} & p^{(k)}_{\mathcal{S}-} \end{bmatrix} \right\}\right) \tag{S14}$$

where $\otimes$ denotes the Kronecker product and s and $s^{\dagger}$ are the predefined similarity and dissimilarity. Abuse of notation is used to represent the construction of $\vec{r}^{(k)}_{\mathrm{ui}}$ as the vectorization of the two matrices in

a row, from top to bottom and from left to right.

Table S3: Variables involved in the evaluation of use intentions in the Silicon Coppélia system, and their corresponding kets in the Quantum Coppélia system.

Name	Si-Coppélia notation	Description	Qubit notation
Expected utility	${u'}_{ij}^{(k)}$	${u'}_{ij}^{(k)} \in [-1, 1]$	$\left\|{u'}_{ij,+}^{(k)}\right\rangle$: *indicative* $\left\|{u'}_{ij,-}^{(k)}\right\rangle$: *counter-indicative*
Mean expected utility over goals	$\bar{u}_{i}'^{(k)}$	$\bar{u}_{i}'^{(k)} \in [-1, 1]$	$\left\|\bar{u}_{i,+}'^{(k)}\right\rangle$: *indicative* $\left\|\bar{u}_{i,-}'^{(k)}\right\rangle$: *counter-indicative*
Indicative & Counter-indicative utility	${u'}_{+}^{(k)}$, ${u'}_{-}^{(k)}$	${u'}_{+}^{(k)}, {u'}_{-}^{(k)} \in [0, 1]$	$\left\|{u'}_{+}^{(k)}\right\rangle$: *indicative* $\left\|{u'}_{-}^{(k)}\right\rangle$: *counter-indicative*
Weight function for ${u'}_{ij}^{(k)}$	$\Theta_{i}^{(k)}\left({u'}_{ij}^{(k)}\right)$	$\sum_{j} \Theta_{j}^{(k)}\left({u'}_{ij}^{(k)}\right) = 1$	Embedded in R_y rotations
Component vector for use intentions	$\vec{r}_{\mathrm{ui}}^{(k)}$	10 components	Formed by constitute qubits as controls
Weight matrix for use intentions	$\mathbf{B}_{\mathrm{ui}}$	2×10 matrix, $\sum_{i} (\mathbf{B}_{\mathrm{ui}})_{ij} \leq 1$	Embedded in R_y rotations
Use intentions (UI)	$\vec{u}^{(k)} = \left(u_{+}^{(k)}, u_{-}^{(k)}\right)^{\mathrm{T}}$	$u_{+}^{(k)}, u_{-}^{(k)} \in [0, 1]$	$\left\|u_{+}^{(k)}\right\rangle$: +ve UI $\left\|u_{-}^{(k)}\right\rangle$: −ve UI

S1.3.2 Engagement as involvement and distance

The robot establishes engagement with an agency by calculating the levels of *involvement* with and *distance* towards, for instance, its user. Involvement and distance are two tendencies that occur in parallel and may compensate for one another. This trade-off in the engagement process is accompanied by specific emotions, which may differ in each particular case. The variables involved in the comparison phase are summarized in Table S4. Similar to use intentions, involvement–distance is modeled as a complex composition of various factors, each carrying a particular weight. The involvement ($E_{\mathrm{inv}}^{(k)}$) and distance

($E_{\text{dist}}^{(k)}$) is given by

$$\vec{E}^{(k)} = \begin{bmatrix} E_{\text{inv}}^{(k)} \\ E_{\text{dist}}^{(k)} \end{bmatrix} = \mathbf{B}_{\text{id}}\, \vec{r}_{\text{id}}^{(k)} \tag{S15}$$

where the 2×26 matrix $\mathbf{B}_{\text{id}}$ is the weight matrix for involvement–distance, containing the weights for $\vec{r}_{\text{id}}^{(k)}$, the component vector in $\mathbb{R}^{26}$:

$$\vec{r}_{\text{id}}^{(k)} = \text{vec}\left(\left\{ \begin{bmatrix} p_{\mathcal{S}+}^{(k)} & p_{\mathcal{P}+}^{(k)} & p_{\mathcal{F}+}^{(k)} & R^{(k)} & V^{(k)} \\ p_{\mathcal{S}-}^{(k)} & p_{\mathcal{P}-}^{(k)} & p_{\mathcal{F}-}^{(k)} & R^{\dagger(k)} & V^{\dagger(k)} \end{bmatrix}, \begin{bmatrix} R^{(k)} & V^{(k)} \\ R^{\dagger(k)} & V^{\dagger(k)} \end{bmatrix} \otimes \begin{bmatrix} p_{\mathcal{S}+}^{(k)} & s \\ p_{\mathcal{S}-}^{(k)} & s^{\dagger} \end{bmatrix} \right\}\right) \tag{S16}$$

where the convention of vectorization is the same as that in Eq. (S13). The involvement–distance trade-off parameter is then calculated as the weighted mean of the *fuzzy-or* operator and the compensation between involvement with distance and the compensation factor for the two (β_{idt}):

$$E_{\text{idt}}'^{(k)} = \beta_{\text{idt}} \max\left(E_{\text{inv}}^{(k)}, E_{\text{dist}}^{(k)}\right) + (1 - \beta_{\text{idt}}) \frac{E_{\text{inv}}^{(k)} + E_{\text{dist}}^{(k)}}{2} \tag{S17}$$

Table S4: Variables in the involvement–distance trade-off of the Silicon Coppélia system, and their corresponding kets in the Quantum Coppélia system.

Name	Si-Coppélia notation	Description	Qubit notation
Component vector for involvement–distance	$\vec{r}_{\text{id}}^{(k)}$	26 components	Formed by constitute qubits as controls
Weight matrix for involvement–distance	$\mathbf{B}_{\text{id}}$	2×26 matrix, $\sum_i (\mathbf{B}_{\text{id}})_{ij} \leq 1$	Embedded in R_y rotations
Involvement–distance	$\vec{E}^{(k)} = \left(E_{\text{inv}}^{(k)}, E_{\text{dist}}^{(k)}\right)^{\text{T}}$	$E_{\text{inv}}^{(k)}, E_{\text{dist}}^{(k)} \in [0, 1]$	$\left\lvert E_{\text{inv}}^{(k)}\right\rangle$: *involvement* $\left\lvert E_{\text{dist}}^{(k)}\right\rangle$: *distance*
Compensation factor for involvement & distance	β_{idt}	$\beta_{\text{idt}} \in [0, 1]$	$\lvert\beta_{\text{idt}}\rangle$

Table S4: Variables in the involvement–distance trade-off of the Silicon Coppélia system, and their corresponding kets in the Quantum Coppélia system. (Cont'd)

Name	Si-Coppélia notation	Description	Qubit notation
Involvement–distance trade-off	$E'^{(k)}_{\mathrm{idt}}$	$E'^{(k)}_{\mathrm{idt}} \in [0,1]$	$\left\lvert E'^{(k)}_{\mathrm{idt}} \right\rangle$

S1.3.3 Satisfaction

Use intentions, involvement, and distance together determine the overall *satisfaction* of the robot with its user or another agency. The level of satisfaction determines the robot's decision of whether and how it is going to interact with the user (or avoid – stop and turn to another). The variables involved in the calculation are summarized in Table S5. The decision-making process is carried out by calculating the *expected satisfaction* of interacting (again) with a user, which is the weighted mean of the involvement-distance trade-off (IDT) and the average of the indicative and counter-indicative use intentions with the respective weights σ'_{idt} and σ'_{ui}:

$$S^{(k)} = \sigma'_{\mathrm{idt}} E'^{(k)}_{\mathrm{idt}} + \sigma'_{\mathrm{ui}} \frac{u_+^{(k)} + u_-^{(k)}}{2}. \tag{S18}$$

Eq. (S18) reveals that the robot makes a decision on the more rationally generated use intentions in unison with the more affectively generated involvement-distance trade-off. The feature $k_{\max}$ that promises the highest expected satisfaction during the interaction is selected, i.e., treating $S^{(k)}$ as a function of k,

$$k_{\max} = \arg\left(\max_{k\in\mathscr{F}}\big(S^{(k)}\big)\right). \tag{S19}$$

After selecting the feature of the highest satisfaction, the robot inspects the overt actions $i^{(k_{\max})}$ available to this feature, and chooses an action tendency (as noted in *relevance* and *valence* in Section S1.2.1) from the following 4 types:

1. positive approach (e.g., compliment the user): $t(i) = \mathrm{p}$

2. negative approach (e.g., criticize the user): $t(i) = \mathrm{n}$

3. change (e.g., teach the user): $t(i) = \mathrm{c}$

4. avoid the user: $t(i) = \mathrm{a}$.

The 4 types of action tendencies are depicted in the report of Silicon Coppélia.[2] In general, a feature may not offer all the 4 action tendencies. Additional action tendencies are also possible (e.g., do nothing).

The selection of the action tendency is done by calculating the respective expected satisfaction upon the action:

$$S_i^{(k_{\max})} = \begin{cases} \sigma_{\mathrm{i,p}} E_{\mathrm{inv}}^{(k_{\max})} + \sigma_{\mathrm{d,p}} \left(1 - E_{\mathrm{dist}}^{(k_{\max})}\right) + \sigma_{\mathrm{u,p}} \bar{u}_i'^{(k_{\max})}, & t(i) = \mathrm{p} \\ \sigma_{\mathrm{i,n}} \left(1 - E_{\mathrm{inv}}^{(k_{\max})}\right) + \sigma_{\mathrm{d,n}} E_{\mathrm{dist}}^{(k_{\max})} + \sigma_{\mathrm{u,n}} \bar{u}_i'^{(k_{\max})}, & t(i) = \mathrm{n} \\ \sigma_{\mathrm{i,c}} E_{\mathrm{inv}}^{(k_{\max})} + \sigma_{\mathrm{d,c}} E_{\mathrm{dist}}^{(k_{\max})} + \sigma_{\mathrm{u,c}} \bar{u}_i'^{(k_{\max})}, & t(i) = \mathrm{c} \\ \sigma_{\mathrm{i,a}} \left(1 - E_{\mathrm{inv}}^{(k_{\max})}\right) + \sigma_{\mathrm{d,a}} E_{\mathrm{dist}}^{(k_{\max})} + \sigma_{\mathrm{u,a}} \bar{u}_i'^{(k_{\max})}, & t(i) = \mathrm{a} \end{cases} \tag{S20}$$

or, in matrix representation,

$$\begin{bmatrix} S_{i|t(i)=\mathrm{p}}^{(k_{\max})} \\ S_{i|t(i)=\mathrm{n}}^{(k_{\max})} \\ S_{i|t(i)=\mathrm{c}}^{(k_{\max})} \\ S_{i|t(i)=\mathrm{a}}^{(k_{\max})} \end{bmatrix} = \begin{bmatrix} \sigma_{\mathrm{i,p}} & -\sigma_{\mathrm{d,p}} & \sigma_{\mathrm{u,p}} & \sigma_{\mathrm{d,p}} \\ -\sigma_{\mathrm{i,n}} & \sigma_{\mathrm{d,n}} & \sigma_{\mathrm{u,n}} & \sigma_{\mathrm{i,n}} \\ \sigma_{\mathrm{i,c}} & \sigma_{\mathrm{d,c}} & \sigma_{\mathrm{u,c}} & 0 \\ -\sigma_{\mathrm{i,a}} & \sigma_{\mathrm{d,a}} & \sigma_{\mathrm{u,a}} & \sigma_{\mathrm{i,a}} \end{bmatrix} \begin{bmatrix} E_{\mathrm{inv}}^{(k_{\max})} \\ E_{\mathrm{dist}}^{(k_{\max})} \\ \bar{u}_i'^{(k_{\max})} \\ 1 \end{bmatrix}. \tag{S21}$$

The action with the highest expected satisfaction will be chosen as the final response, i.e., treating $S_i^{(k_{\max})}$ as a function of $i^{(k_{\max})}$,

$$i_{\max} = \arg\left(\max_{i \in \mathscr{A}^{(k_{\max})}} \left(S_i^{(k_{\max})} \right) \right). \tag{S22}$$

Table S5: Variables in the satisfaction evaluation of the Silicon Coppélia system, and their corresponding kets in Quantum Coppélia.

Name	Si-Coppélia notation	Description	Qubit notation
Weight factors for expected satisfaction	$\sigma'_{\mathrm{idt}}, \sigma'_{\mathrm{ui}}$	$\sigma'_{\mathrm{idt}}, \sigma'_{\mathrm{ui}} \in [0, 1]$	Embedded in R_y rotations

Table S5: Variables in the satisfaction evaluation of the Silicon Coppélia system, and their corresponding kets in Quantum Coppélia. (Cont'd)

Name	Si-Coppélia notation	Description	Qubit notation
Expected satisfaction	$S^{(k)}$	$S^{(k)} \in [0,1]$	$\left\|S^{(k)}\right\rangle$
Feature contributing to the greatest expected satisfaction	$k_{\max}$	$k_{\max} \in \mathscr{F}$	$\|k_{\max}\rangle$
Component weight for expected satisfaction for action $i^{(k)}$	$\sigma_{\mu,\nu}$	$\mu = \mathrm{i,d,u};\ \nu = \mathrm{p,n,c,a}$ $\sum_{\mu} \sigma_{\mu,\nu} \leq 1$	Embedded in R_y rotations
Expected satisfaction for action $i^{(k)}$	$S_i^{(k)}$	$S_i^{(k)} \in [-1,1]$	$\left\|S_i^{(k)}\right\rangle$
Action contributing to the greatest expected satisfaction $S_i^{(k_{\max})}$	$i_{\max}$	$i_{\max} \in \mathscr{A}^{(k_{\max})}$	$\|i_{\max}\rangle$

References

[1] Van Vugt, H. C., Hoorn, J. F. & Konijn, E. A. Interactive engagement with embodied agents: An empirically validated framework. *Computer Animation and Virtual Worlds* **20,** 195–204; https://doi.org/10.1002/cav.312 (2009).

[2] Hoorn, J. F., Baier, T., Van Maanen, J. A. N. & Wester, J. Silicon Coppélia and the formalization of the affective process. *IEEE Trans. Affect. Comput.*; https://doi.org/10.1109/TAFFC.2020.3048587 (2021).
